# Genetic mapping for agronomic traits in a MAGIC population of common bean (*Phaseolus vulgaris L.)* under drought conditions

**DOI:** 10.1186/s12864-020-07213-6

**Published:** 2020-11-16

**Authors:** Santiago Diaz, Daniel Ariza-Suarez, Paulo Izquierdo, Juan David Lobaton, Juan Fernando de la Hoz, Fernando Acevedo, Jorge Duitama, Alberto F. Guerrero, Cesar Cajiao, Victor Mayor, Stephen E. Beebe, Bodo Raatz

**Affiliations:** 1 Bean Program, Agrobiodiversity Area, International Center for Tropical Agriculture (CIAT), Cali, Colombia; 2 Present Address: Department of Plant Soil and Microbial Sciences, Michigan State University, East Lansing, MI USA; 3 Present Address: School of Environmental and Rural Sciences, University of New England, Armidale, SA Australia; 4 Present Address: Bioinformatics Interdepartmental Ph.D. Program, University of California, Los Angeles, Los Angeles, CA USA; 5 Departamento de Agronomía, Facultad de Ciencias Agrarias, Universidad Nacional de Colombia, Bogotá, Colombia; 6 Present Address: Systems and Computing Engineering Department, Universidad de los Andes, Bogotá, Colombia; 7 Present Address: Progeny Breeding, Madrid, Colombia

**Keywords:** Genotyping-by-sequencing (GBS), Genome-wide association study (GWAS), Multiparent advanced generation inter-crosses (MAGIC), Quantitative trait loci (QTL), Whole genome sequencing (WGS)

## Abstract

**Background:**

Common bean is an important staple crop in the tropics of Africa, Asia and the Americas. Particularly smallholder farmers rely on bean as a source for calories, protein and micronutrients. Drought is a major production constraint for common bean, a situation that will be aggravated with current climate change scenarios. In this context, new tools designed to understand the genetic basis governing the phenotypic responses to abiotic stress are required to improve transfer of desirable traits into cultivated beans.

**Results:**

A multiparent advanced generation intercross (MAGIC) population of common bean was generated from eight Mesoamerican breeding lines representing the phenotypic and genotypic diversity of the CIAT Mesoamerican breeding program. This population was assessed under drought conditions in two field trials for yield, 100 seed weight, iron and zinc accumulation, phenology and pod harvest index.

Transgressive segregation was observed for most of these traits. Yield was positively correlated with yield components and pod harvest index (PHI), and negative correlations were found with phenology traits and micromineral contents. Founder haplotypes in the population were identified using Genotyping by Sequencing (GBS). No major population structure was observed in the population. Whole Genome Sequencing (WGS) data from the founder lines was used to impute genotyping data for GWAS. Genetic mapping was carried out with two methods, using association mapping with GWAS, and linkage mapping with haplotype-based interval screening. Thirteen high confidence QTL were identified using both methods and several QTL hotspots were found controlling multiple traits. A major QTL hotspot located on chromosome Pv01 for phenology traits and yield was identified. Further hotspots affecting several traits were observed on chromosomes Pv03 and Pv08. A major QTL for seed Fe content was contributed by MIB778, the founder line with highest micromineral accumulation. Based on imputed WGS data, candidate genes are reported for the identified major QTL, and sequence changes were identified that could cause the phenotypic variation.

**Conclusions:**

This work demonstrates the importance of this common bean MAGIC population for genetic mapping of agronomic traits, to identify trait associations for molecular breeding tool design and as a new genetic resource for the bean research community.

**Supplementary Information:**

The online version contains supplementary material available at 10.1186/s12864-020-07213-6.

## Background

Common bean (*Phaseolus vulgaris* L.) is one of the most important grain legumes for direct human consumption [1], and it is widely cultivated throughout the world, especially in tropical and subtropical countries of Africa and America [2]. Bean has been recognized as a highly valuable food for human nutrition, a rich and relatively inexpensive source of proteins, micronutrients such as iron and zinc, dietary fiber and essential vitamins [3] making it valuable food for over half a billion people, especially in developing countries [4]. Micronutrient malnutrition (MNM) is considered a major health threat affecting half the world’s population, particularly women and children in developing countries [5]. Iron deficiency anemia, which is the most prevalent MNM in the world, can be alleviated by biofortification, particularly in legumes with high baseline Fe contents such as common bean [6].

Bean production is negatively affected by multiple abiotic and biotic constraints. Drought is a major factor to yield loss around the world and its incidence and duration is expected to increase due to climate change [4, 7]. It is estimated that approximately 60% of common bean production is affected by terminal or intermittent drought [8]. If current climate change trends continue, a large part of current common bean growing areas in southeastern Africa are predicted to become unsuitable for bean cultivation by 2050, generating reductions in yield for current varieties and potentially affecting the nutritional quality of the crop [9]. In that sense, drought represents a high-risk factor for bean farmers in the tropics, and improved varieties developed to withstand altered climatic conditions represent a valuable resource for future generations.

Genetic mapping is used to identify genomic regions that harbor variation responsible for altering phenotypic trait expression. This information is then applied in breeding by marker-assisted selection (MAS), whereby breeding lines with desirable trait attributes are identified through genetic screening. Different strategies are available for genetic mapping. Most molecular markers currently used in breeding were identified through linkage mapping, where a bi-parental population is generated for identifying the genomic regions that segregate with a trait. However, this strategy has low resolution because of limited genetic recombinations and limited genetic variation, since only two haplotypes are observed per locus [10]. On the other hand, association mapping using Genome-wide association studies (GWAS) directly identifies marker-trait associations in diverse populations. This strategy also leverages low levels of linkage disequilibrium (LD) to achieve higher resolution. However, confounders such as population structure can produce spurious associations, and large populations are needed to overcome the lack of statistical power in the case of low allele frequencies [11].

A strategy to increase the number of investigated haplotypes while avoiding confounding population structure effects is to generate recombinant inbred lines (RILs) from multiple parents [12], where the genomes of the founders are first recombined through several rounds of mating and then advanced to generate a stable panel of inbred lines. The creation of Multiparent advanced generation intercross (MAGIC) populations holds great promise for genetic mapping, overcoming the main limitations of linkage and association mapping [13]. Using more than two parental accessions increases the allelic and phenotypic diversity compared to traditional bi-parental populations, raising the number of QTL that segregate in the population and the larger number of accumulated recombination events increases mapping accuracy [14]. Furthermore, MAGIC populations have been suggested to use breeding lines as founders, rather than conventional crosses between two phenotypically contrasting lines. Utilization of elite breeding germplasm in MAGIC population development strongly facilitates the transfer of identified QTL into breeding applications. In summary, a MAGIC population combines advantages of natural and synthetic populations with a better shuffling of the genome and increased genetic resolution (i.e. better QTLs) and better transferability to breeding applications [15].

Genetic mapping ideally leads to the identification of genes, whose functional alleles influence the observed phenotypic variation. Gene identification is most advanced in model crops like rice, where over 2000 genes controlling important agronomic traits have been cloned [16, 17]. There are few examples for cloned genes in common bean, including the *bc-3* gene conferring resistance to bean common mosaic virus, which was identified as the *eIF4E* gene [18] or *Mrp1* gene, causative of *lpa1* mutant regulating low concentration of phytic acid in the bean seed [19]. In other studies, candidate genes related with agronomic traits in common bean have been identified based on annotations in the vicinity of significantly associated markers [20–23]. Further investigation of candidate genes is hindered by the scarcity of genetic resources and genetic transformation protocols in common bean [24], which would be required for validation experiments.

The main goal of this work was to develop a common bean MAGIC population from eight Mesoamerican elite breeding founder lines and to identify genomic regions associated with yield, micromineral accumulation, phenology and physiological traits under drought conditions.

## Results

### Phenotypic evaluation

A common bean MAGIC population was developed by inter-crossing of eight Mesoamerican elite breeding lines, including four founder lines derived from interspecific crosses with *P. coccineus*, *P. dumosus* and *P. acutifolius* (Table 1). Following the crossing scheme in Additional file 1, 996 RILs were generated. Field trials under drought stress were carried out in 2013 and 2014 (Additional file 2) with 636 and 599 RIL lines respectively. Agronomic performance was evaluated with respect to yield (Yd), days to flowering (DF), days to physiological maturity (DPM), seed weight (100SdW) and pod harvest index (PHI). Micronutrient contents such as iron and zinc in the seed (SdFe, SdZn) were evaluated in 2014 and again in non-stress conditions in 2016.

**Table 1:** Description of the eight common bean (*P. vulgaris*) founder lines of the MAGIC population

Line	Interspecific introgressions from	Pedigree	Valuable traits
SXB412		A686/A774//NXB80/SEA15	Low fertility and drought tolerance
INB827	*P. acutifolius*	INB108/INB605	Drought tolerance
ALB213	*P. coccineus*	SER16/G35346-3Q//SER16	Drought and Al tolerance
SEN56		SXB123/DOR677//SEN34	Drought tolerance
SCR2		NCB226/RCB591	Drought tolerance and BCMV^a^ resistance
MIB778	*P. dumosus*	FEB226/G35575-2P//FEB226	High Fe/Zn seed content
SCR9		SER176/RCB591	Drought tolerance and BCMV^a^ resistance
INB841	*P. acutifolius*	INB108/INB605	Drought tolerance

^a^*BCMV* bean common mosaic virus

**Fig. 1:**
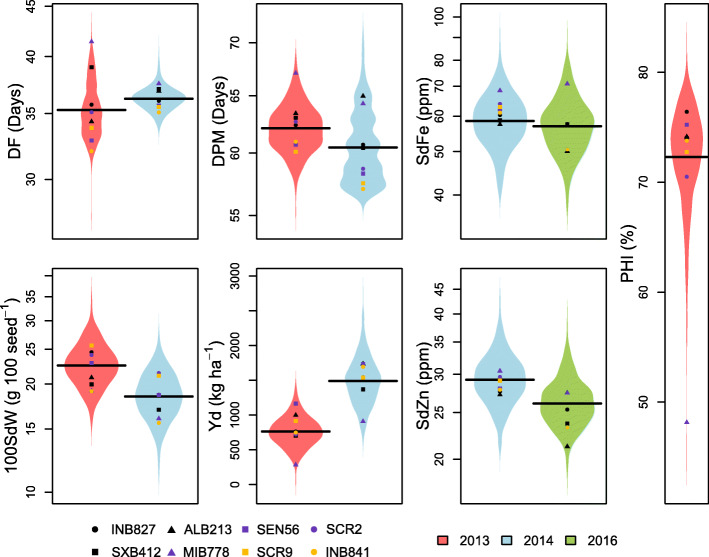
Distribution of best linear unbiased estimators (BLUEs) of the evaluated traits in the trials of 2013, 2014 and 2016 for the MAGIC population. DF: Days to flowering; 100SdW: 100 seed weight; DPM: Days to physiological maturity; Yd: Seed yield; SdFe: Seed iron; SdZn: Seed Zinc; PHI: Pod harvest index

**Fig. 2:**
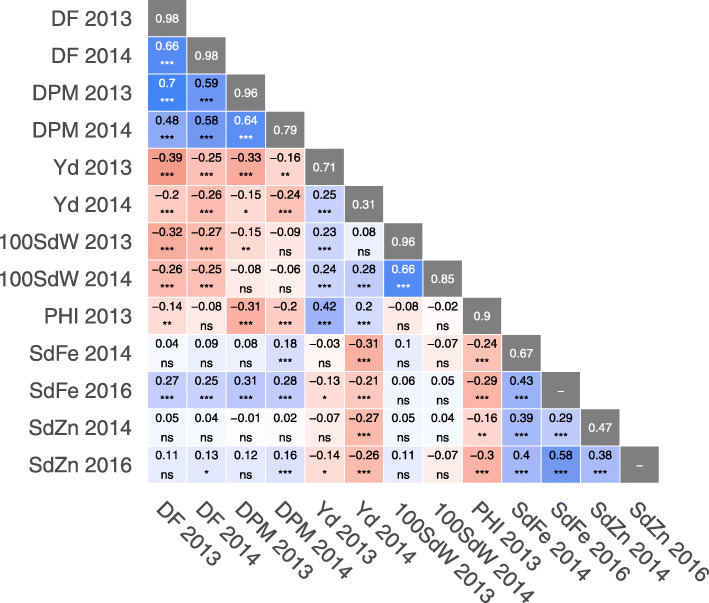
Pearson’s correlation coefficients between best linear unbiased estimators (BLUEs) of evaluated traits. The broad-sense heritabilities of the best linear unbiased predictors (BLUPs) are located within the diagonal with gray background. Significance of correlations indicated as ***: p < .0001; **: p < .001; *: p < .01; ns = not significant. DF: Days to flowering; 100SdW: 100 seed weight; DPM: Days to physiological maturity; Yd: Seed yield; SdFe: Seed iron; SdZn: Seed Zinc; PHI: Pod harvest index

The phenotypic data display significant genetic variability within all traits, and transgressive segregation was observed for most of them (Fig. 1). The broad-sense heritability for phenological traits was high, with 0.98 for DF in both years and 0.96 and 0.79 respectively for DPM (Fig. 2). PHI had a heritability of 0.90. Likewise, high heritabilities of 0.96 and 0.85 were observed for 100SdW in respective years. In contrast, the heritability for yield was lower and not stable across both trials with 0.71 in 2013 and 0.31 in 2014. Heritabilities of iron and zinc content were intermediate, with 0.67 for SdFe and 0.47 for SdZn. In general, trait heritabilities in 2013 were higher compared to 2014 for all traits measured in both trials. In 2013, two-row plots were used compared to one-row plots in 2014, which may have caused more experimental noise, allowing more precise data in 2013. However, climatic conditions differed between seasons with stronger drought and less late rainfall in 2013 (Additional file 2), leading to a stronger yield reduction.

In spite of the seasonal variation, all traits measured in more than one trial were significantly positively correlated, ranging from 0.25 (Yd) to 0.66 (DF and 100SdW) (Fig. 2), indicating comparability of the data sets from the three trials for evaluated traits. Two trait clusters were identified according to the positive and generally significant correlations within the clusters and negative correlations between them. On the one hand, the phenology traits DF and DPM and micronutrient contents SdFe and SdZn form a group of traits that were largely positively correlated to each other. On the other hand, yield and 100SdW together with PHI show significant positive correlations among them, while being negatively correlated to traits of the first group (Fig. 2). The founders SCR2 and SCR9 performed best in terms of yield and related traits, while MIB778 showed delayed maturity, lowest yield components, but the highest Fe and Zn contents (Fig. 1 and Additional file 3). Taken together, the phenotypic datasets from the field trials show a good quality for subsequent analyses, with some lines showing transgressive segregation and outperforming elite founder lines.

### Genetic and population structure

**Fig. 3:**
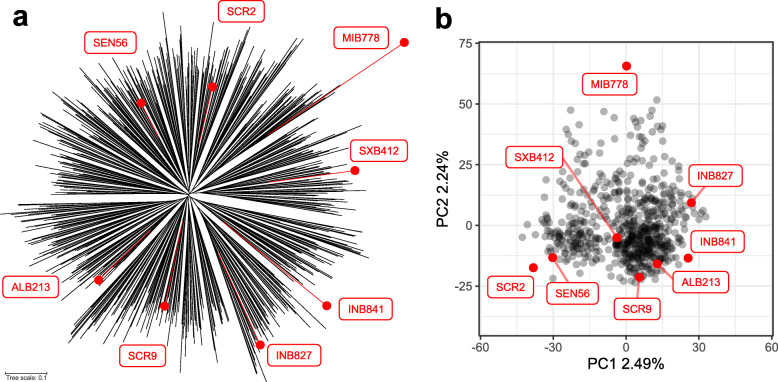
Assessment of population structure for 629 MAGIC lines and 8 founders using GBS data (20.615 markers). **a** Unrooted neighbour-joining tree. The length of the lines in the tree show the simple matching distance. **b** Location of each genotype represented by a point in the two-dimensional space defined by the eigenvectors of the first and second principal components. The founder lines are represented by red tagged points

The MAGIC population was genotyped by GBS, generating 20,615 polymorphic markers that were used to assess the population structure. A binning process to eliminate redundant markers was applied, resulting in 5738 non-redundant markers that were used to generate a genetic map and for QTL mapping. WGS data from the eight founder lines was used to impute 1,972,528 markers in the population for GWAS (Additional files 4 and 5). The population structure was assessed by constructing a NJ tree and performing a PCA. All MAGIC and founder lines are evenly separated from the center of the tree (Fig. 3a), except for MIB778 which showed a longer matching distance. There are no defined clusters that separate lines in the tree, indicating no significant population structure within the MAGIC population. In line with these results, each principal component explains only a small proportion of the variance, as the first two principal components account just for 4.73% (Fig. 3b). The two-dimensional space defined by the first and second principal components show a uniform dispersal of the genotypes, with MIB778 drawing more distant from the other founders. This result mirrors the actual pedigree, since MIB778 has the least number of common ancestors with other founder lines (Additional file 6). These results indicate that within the MAGIC lines the population structure has a low level of complexity.

**Fig. 4:**
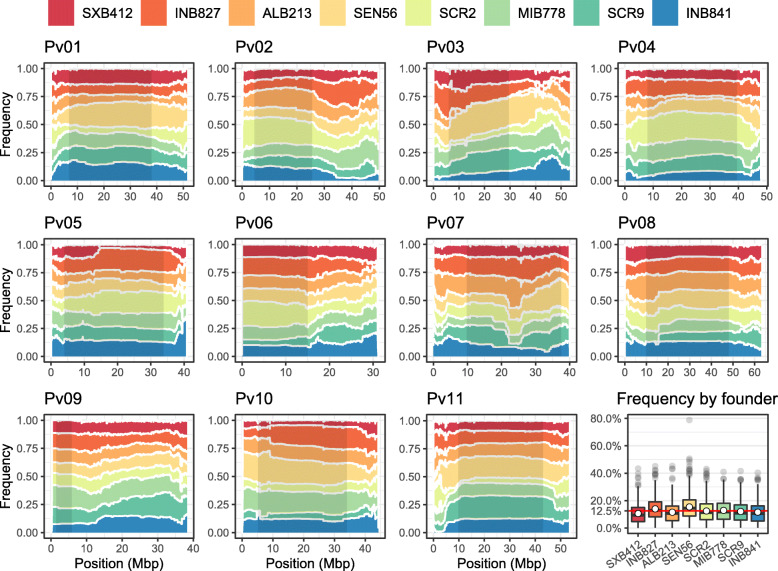
Distribution of the founder’s haplotypes on each chromosome in the MAGIC population. The inner black-shaded region represents the boundaries for the pericentromeric regions. Overall founders’ contribution on the 629 genotyped MAGIC lines genome are indicated in the bottom-right boxplot. The expected value of 12.5% (1/8th) is indicated by a red line

A haplotype analysis was performed to quantify the individual contribution from each founder line in the population. The average fraction of each founder on the MAGIC lines was similar, ranging between 10.6 and 15.4%. These values were close to the expected contribution of 1/8th (12.5%). However, this fraction was not constant along the chromosomes, especially in the euchromatic regions (Fig. 4), which may be attributed to arbitrary segregation after the early crosses that were performed in a limited number of plants. The predicted haplotype composition for each RIL is presented in Additional file 7. Taken together, the reduced complexity level of population structure and an even representation of parental haplotypes in the MAGIC lines display the intended characteristics of the population to perform genetic analyses.

A genetic map for the MAGIC population was generated with a total length of 857 cM. The recombination rate was 2407 kbp/cM in the pericentromeric regions and 291 kbp/cM in the euchromatic regions of the chromosomes, with an overall rate of 603 kbp/cM. The MAGIC population had a mean of 3.22 recombination events per chromosome and a median of 44 recombination events per RIL in the entire genome (Additional files 7 and 8). The genome-wide rate of LD decay was 74 kbp at 0.19 ${r}_V^2$, about half of its initial predicted value. The rate of chromosome-specific LD decay ranged between 51 kbp (Pv08) and 154 kbp (Pv02) (Additional file 9). The high marker density and accuracy due to the large population size make this map suitable for the analysis of linkage and segregation patterns and for genetic mapping.

### QTL analysis and genome-wide association study

Marker-trait associations were evaluated by both GWAS, using a Mixed Linear Model (MLM) approach, and QTL mapping, using software designed for haplotype-based analysis of multi-parent populations. In total, 17 QTL for 7 traits were identified with significant peaks in the GWAS analysis (Additional file 10). GWAS peaks were selected with a significance greater than that established by the Bonferroni correction (2.42 × 10^− 6^) and selecting those regions that showed a clear peak above the background. QTL analysis resulted in 45 QTL for 7 traits exceeding the threshold of significance set by 1000 permutations (LOD > 6.26) (Additional file 11). High confidence QTL were defined as those showing significant marker-trait associations in both GWAS and QTL mapping. Following that rule, thirteen such “main QTL” were identified for 7 traits (Table 2). Founder haplotype assignment of phenotypic effects was generally the same between GWAS and QTL analysis (Additional files 10 and 11).

**Table 2:** Major QTL in the MAGIC population identified in both MLM-based GWAS and QTL analysis optimized for complex populations. QTL are listed that were significant in both analyses and show clear peaks in GWAS. A complete list of significant marker trait associations is available for GWAS (Additional file 10) and QTL mapping (Additional file 11)

QTL name	Trial	GWAS				QTL Mapping			
		Most significant marker^a^	***p*** value	MAF	Effect^b^	Pos. (cM)	Nearest marker	LOD	PVE (%)^c^
*DF1.1*	2013	Pv2.1_01_4147947_C/G	5.46E-07	0.37 (G)	−0.46	32	Pv2.1_01_5474208_C/T	9.30	4.30
*DPM1.1*	2013	Pv2.1_01_763904_G/A	1.60E-06	0.27 (A)	0.43	36.5	Pv2.1_01_6773375_T/A	21.70	15.90
*DF1.2*	2013	Pv2.1_01_14672594_C/T	1.05E-35	0.34 (C)	1.63	38	Pv2.1_01_11238077_T/A	58.85	35.76
	2014	Pv2.1_01_17627372_A/G	2.36E-12	0.35 (G)	−0.28	40	Pv2.1_01_14582969_G/C	34.42	16.50
*DPM1.2*	2013	Pv2.1_01_14672594_C/T	2.77E-09	0.34 (C)	0.82	38.5	Pv2.1_01_13085941_C/A	8.19	5.47
	2014	Pv2.1_01_11567887_T/TA	2.13E-07	0.18 (T)	0.61				
*Yd1.1*	2013	Pv2.1_01_11250640_A/G	3.11E-08	0.16 (A)	−52.81	38	Pv2.1_01_11238077_T/A	8.76	2.03
	2014	Pv2.1_01_11221702_T/A	3.57E-07	0.21 (T)	−28.79				
*PHI2.1*	2013	Pv2.1_02_47566148_G/A	2.61E-08	0.37 (G)	−1.08	71	Pv2.1_02_47643879_G/T	10.73	7.62
*DF3.1*	2013	Pv2.1_03_39652029_G/A	5.23E-07	0.14 (A)	0.62	50	Pv2.1_03_39061112_G/C	7.50	3.43
	2014					50	Pv2.1_03_39061112_G/C	8.11	3.54
*DPM3.1*	2013	Pv2.1_03_39534987_C/A	2.08E-08	0.16 (C)	−0.75	48	Pv2.1_03_37341462_T/C	11.41	8.36
	2014					51.5	Pv2.1_03_39819948_G/A	10.24	5.11
*100SdW4.1*	2014	Pv2.1_04_1328790_G/A	4.08E-08	0.27 (A)	0.51	3	Pv2.1_04_522512_A/T	8.36	4.74
*SdFe6.1*	2014	Pv2.1_06_5311627_C/T	2.77E-07	0.17 (C)	2.09	2	Pv2.1_06_8725963_G/A	7.33	4.06
*SdFe6.2*	2016	Pv2.1_06_22844368_T/G	3.36E-07	0.08 (G)	−2.67	35	Pv2.1_06_23945432_T/C	6.46	8.68
*DPM8.1*	2013	Pv2.1_08_1070342_C/T	2.54E-09	0.11 (T)	−1.45	5	Pv2.1_08_1091562_T/G	7.92	5.56
	2014	Pv2.1_08_706882_A/G	3.58E-11	0.13 (G)	−1.77	4	Pv2.1_08_946243_C/T	18.15	9.09
*SdZn8.1*	2014	Pv2.1_08_60922896_C/G	4.39E-07	0.06 (C)	0.85	83	Pv2.1_08_60982396_C/G	6.81	2.65

^a^Marker ID contains the physical position of the polymorphism in the reference version 2.1 and the genotype of the reference / alternative allele

^b^Effect of alternative allele

^c^PVE = Phenotypic variation explained

Most significant trait-associations were identified on chromosome Pv01 for phenology traits DF and DPM, in the region between 10 and 18 Mbp (Table 2). This region showed additive effects of 0.1–1.7 days and 0.6–0.8 days for *DF1.2* and *DPM1.2* respectively. *DF1.2* and *DPM1.2* explained 35.8 and 5.5% of the phenotypic variance, respectively. A yield QTL was found in the same genomic region (*Yd1.1*), with phenotypic effects of up to 53 kg ha^− 1^. This genomic region represents a QTL hotspot with variation for several traits, as a significant GWAS association was identified also for SdZn (Additional file 10). Evaluating founder allele patterns at this QTL hotspot revealed that between 10.4 and 17.7 Mbp the alleles from SXB412, INB827 and MIB778 had a positive effect on phenology traits. In line with the negative correlation between Yd and phenology traits, alleles from these founder lines had a negative effect on Yd, (Additional file 10). QTL analysis results are mostly in accordance with GWAS results; SXB412, INB827 and MIB778 haplotypes always positively affected phenology traits at this locus. *DF1.2* evaluated in 2013 presented some deviations where SCR2 founder haplotype also had a strong positive effect (Additional file 11). *DF1.2/DPM1.2* showed seasonal stability being detected by GWAS and QTL analysis in both 2013 and 2014 trials. *Yd1.1* was detected at this locus in 2013; however, in 2014 a subtle QTL peak was visible but did not reach the significance threshold (Additional file 12).

Another QTL hotspot for maturity was observed on chromosome Pv03 at 37.3–39.9 Mbp (*DPM3.1 and DF3.1*). In this region, the alleles of INB827 and INB841 had a negative allelic effect on DPM and DF. Similarly, on chromosome Pv06, between 4.4–9.1 Mbp, MIB778 alleles were associated with late maturity and low PHI. MIB778 was the latest flowering founder genotype; accordingly, it was involved in all three QTL on Pv01, Pv03 and Pv06.

A fourth QTL hotspot for maturity and PHI was identified on Pv08 (0–1.6 Mbp). The alleles from the founder line ALB213 were associated with later physiological maturity in both years and low PHI. An interspecific introgression in ALB213 from *P. coccineus* was reported in this same region on the intervals 881,865–884,647 and 1,181,015 to 1,234,070 bp [25], that may cause this phenotype (Table 2, Additional files 10 and 12). In all four QTL hotspots for maturity and yield component traits, defined haplotypes increase maturity days and at the same time decrease yield-related traits, following the negative phenotypic correlations of these trait groups. An interesting QTL *PHI2.1* was found on chromosome Pv02. The alleles of the founder lines SCR2 and SCR9 have a negative effect of − 1.08% on this trait (Additional file 10). This QTL was not linked to maturity traits, for this, it may be easier to employ *PHI2.1* in breeding.

Three main QTL for micromineral content were identified, *SdFe6.1*, *SdFe6.2* and *SdZn8.1* (Table 2). *SdFe6.2* at 21–24 Mbp was observed in two seasons (2014 by GWAS and 2016 by QTL analysis). MIB778 is the founder line with highest accumulation of Fe/Zn and provided the positive alleles for SdFe and SdZn, with an effect of 2.67 ppm in GWAS and 3.10 ppm in QTL analysis (Table 2, Additional files 10 and 11). *SdZn8.1* was located at 60–63 Mbp with an additive allelic effect of 0.85 ppm in GWAS and 0.92 ppm in QTL analysis, also contributed by MIB778 (Table 2). Micromineral levels were evaluated in drought and non-drought conditions to identify possible constitutive QTL. However, most QTL of Fe/Zn were not consistent between the two years of evaluation, which may be due to specific genotype by environment (GxE) effects of drought stress. QTL analysis indicated several further independent QTL that were not detected by GWAS analysis. Among those, two major QTL for yield were located in Pv07 and Pv08 (*Yd7.1* and *Yd8.2*), which showed highly significant LOD scores (23.16 and 38.17 respectively). However, GWAS analysis showed no signal at these regions to validate those results (Additional files 10, 11 and 12).

Top 10 best performing lines were identified based on a weighted trait index (WTI). These lines show an enrichment of desirable haplotypes for major QTL, but no line holds positive alleles for all QTL as these don’t explain the majority of phenotypic variation (Additional files 13 and 14). An outstanding line, MGC583 presents high yield in drought conditions in both trials (1100 and 1600 kg ha^− 1^) and a high accumulation of Fe and Zn (69 and 31 ppm respectively) (Additional file 13).

### Candidate genes

**Fig. 5:**
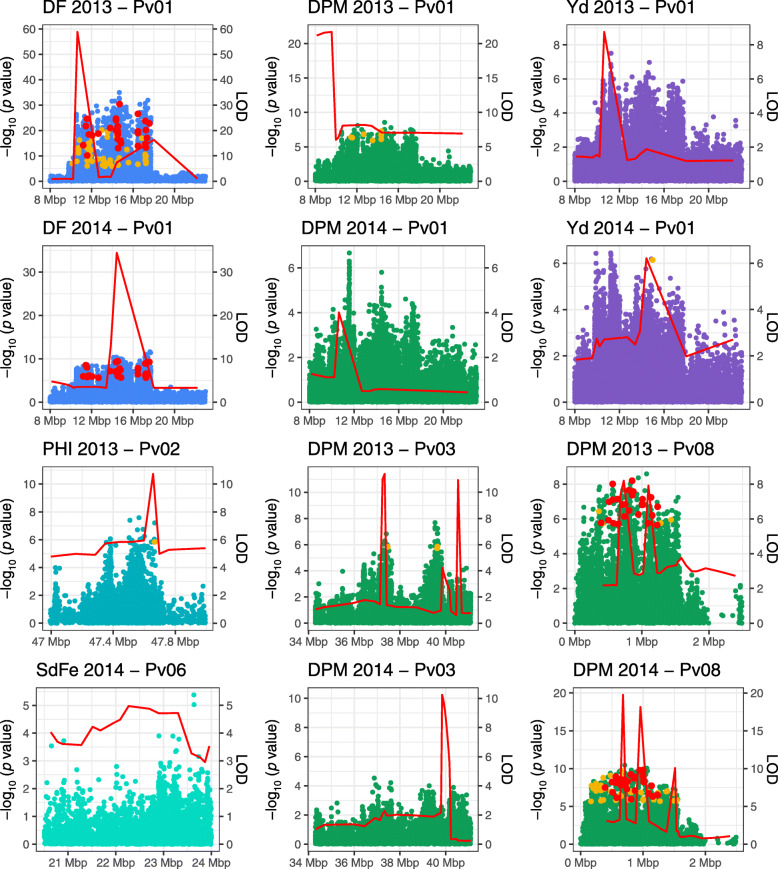
Combined Manhattan plots (GWAS) and LOD (red line obtained from interval mapping) plots of the main QTL regions for DF, DPM, Yd, PHI and SdFe on chromosomes Pv01, Pv02, Pv03, Pv06, Pv08, in the trials of 2013 and 2014. Non-synonymous SNPs in coding regions are highlighted in yellow (significant in only one trial) or red (significant in both trials). Traits are color-coded for improved clarity

GWAS and QTL analysis was performed using WGS data, based on imputation from re-sequenced founder lines. Hence, in principle all SNPs and small indels (< 20 bp) present in the population were evaluated in the GWAS. Close-ups of major QTL regions reveal that QTL analysis LOD curves and GWAS peaks often do not mark the exact same positions (Fig. 5 and Additional file 14); hence, candidate genes were considered based mainly on GWAS analysis data.

To identify candidate genes, only the polymorphisms in coding regions that had a moderate or high effect on the amino acid sequence (as defined by the SnpEff software, see Methods for details) were evaluated. Most candidate polymorphisms were found for the QTL hotspot on Pv01, which had the highest marker-trait associations, and covered the largest genomic region among the major QTL (Table 3). For *DF1.2*, 86 polymorphisms altering the protein sequences of 45 genes were identified and 8 SNPs in 4 genes are shared with *DPM1.2* (Fig. 5 and Additional file 15).

**Table 3:** Candidate genes for the major QTL identified in the MAGIC population. Genes are shown that harbor non-synonymous polymorphisms which had significant associations in GWAS. Most likely candidate genes for major QLT are shown, a complete list of candidate polymorphisms and genes in Additional file 15

Gene	Chr	QTL	Position (bp)^**a**^	Variant (# polymorphisms)	Gene annotation
*Phvul.001G077300*	Pv01	*DF1.2 - DPM1.2*	11,441,607	missense variant (1)	26S proteasome regulatory subunit N6
*Phvul.001G077700*	Pv01	*DF1.2*	11,571,295	splice region (2)	L-arabinokinase
*Phvul.001G085200*	Pv01	*DF1.2*	13,124,249	missense variant (1)	Light regulated protein Lir1
*Phvul.001G087100*	Pv01	*DF1.2 - DPM1.2*	13,513,228	missense variant (1)	GRAM domain (GRAM)
*Phvul.001G087300*	Pv01	*DF1.2*	13,548,191	missense variant (1)	Zinc finger protein CONSTANTS-like 14-related
*Phvul.001G089400*	Pv01	*DF1.2 - DPM1.2*	14,352,260	missense variant (5)	PPR repeat (PPR)
*Phvul.001G089900*	Pv01	*DF1.2*	14,661,652	missense variant (1)	GRAS domain family (GRAS)
*Phvul.001G090100*	Pv01	*DF1.2 - DPM1.2*	14,694,391	missense variant (1)	Exocyst complex protein EXO70
*Phvul.001G091400*	Pv01	*DF1.2*	15,553,393	missense variant (1)	Squamosa promoter-binding protein-like (SBP domain)
*Phvul.001G094300*	Pv01	*DF1.2*	16,832,664	missense variant (1)	SWIB/MDM2 domain
*Phvul.001G093700*	Pv01	*DF1.2*	17,339,464	missense variant (1)	Copine - DCD domain protein
*Phvul.002G309200*	Pv02	*PHI2.1*	47,667,592	missense variant (2)	AGAMOUS-like 29
*Phvul.003G158500*	Pv03	*DPM3.1*	37,464,971	missense variant (1)	Chitinase-like protein-related
*Phvul.006G114800*	Pv06	*SdFe6.2*	22,340,161	missense variant (1)	MYB-like DNA-biding protein MYB4
*Phvul.008G003500*	Pv08	*DPM8.1*	325,238	missense variant (3)	Protein NRT1/PTR family
*Phvul.008G005500*	Pv08	*DPM8.1*	512,002	missense variant (2)	LOB domain-containing protein 40
*Phvul.008G006800*	Pv08	*DPM8.1*	640,209	premature start codon variant (1)	Transducin family protein / WD-40
*Phvul.008G010800*	Pv08	*DPM8.1*	946,243	splice donor variant (1)	Dolichyl-phosphate beta-glucosyltransferase
*Phvul.008G012100*	Pv08	*DPM8.1*	1,001,427	missense variant (4)	transcription initiation factor TFIID subunit 6
*Phvul.008G013300*	Pv08	*DPM8.1*	1,091,953	missense variant (1)	subtilase family protein
*Phvul.008G014000*	Pv08	*DPM8.1*	1,154,068	missense variant (1)	WUSCHEL-related homeobox 10-related

The QTL hotspot on Pv01 contains polymorphisms in genes directly or indirectly involved in flowering pathways. *Phvul.001G085200* encodes a Light Regulator Protein (*Lir1*), a homolog of the perennial ryegrass gene *LpLIR1*, that was reported to be involved in floral induction affecting the control of the circadian clock and vernalization [26]. *Phvul.001G087300* is a homolog of an Arabidopsis B-box zinc finger protein CONSTANS-like 9. CONSTANS and CONSTANS-like genes were reported in Arabidopsis as important regulators of flowering in response to inductive photoperiods, as a central component of photoperiodic floral control [27]. The BBX24 zinc finger transcription factor was also reported to be associated with flowering time and abiotic stress tolerance by repressing flowering and Gibberellin biosynthesis genes in Chrysanthemum [28]. *Phvul.001G089900* is a homolog of the repressor of the gibberellin signaling pathway *RGL1*. This gene is involved in modulating floral development in Arabidopsis [29]. *Phvul.001G094300* is a homolog of Arabidopsis *BAF60,* a SWI/SNF subunit mediating ATP-dependent chromatin remodeling and directly regulating floral repressor FLOWERING LOCUS C [30]. One missense SNP for *Yd1.1* was found; however, annotation information is inconclusive as to regard it as a candidate gene. A short list of 10 most probable candidate genes for the QTL hotspot on Pv01 based on this evaluation is shown in Table 3. Given the large number of genes, a prime candidate cannot be pointed out.

In the QTL hotspot located on Pv03 for DPM and PHI, four polymorphisms in four genes were found. The annotations of these genes suggest their involvement in signaling or developmental processes, but a single candidate gene cannot be clearly attributed to the phenotype.

For the QTL hotspot for DPM and PHI on Pv08, 81 non-synonymous polymorphisms in 51 genes were found, two of which are shared between both traits. Several genes are involved in signaling or development, but the large number makes it difficult to determine a single clear candidate for phenology (Additional file 15). The most significant GWAS association in this QTL was found in *Phvul.008G012100*, next to three further non-synonymous SNPs found in that gene. This gene encodes a TATA BOX ASSOCIATED FACTOR II homolog, a group of genes reported to affect many regulatory processes [31]. A further candidate *Phvul.008G003500* encodes a protein of the NRT1/PTR family. These proteins were originally identified as nitrate or peptide transporters, and recently they have been reported to be transporters of auxins, ABA, and gibberellins [32]. *Phvul.008G014000* is a WUSCHEL-related homeobox 10-related (*WOX*) gene. *WOX* overexpressing lines have been reported with altered flowering times [33]. For *PHI2.1* two non-synonymous polymorphisms were identified in *Phvul.002G309200,* which encodes an AGAMOUS-like gene, a family reported to control development of flowers and fruits [34].

For *SdFe6.2* located on Pv06, four genes were identified with non-synonymous polymorphisms, including *Phvul.006G114800*, which encodes a MYB domain protein 4 homolog (Table 3). MYB-like transcription factors have been shown to affect iron deficiency in *Malus xiaojinensis* [35] or *Chlamydomonas reinhardtii* [36]*,* among multiple other metabolic and developmental processes [37]. The ectopic expression of the *DwMYB2* transgene in *A. thaliana*, a distant homolog of *Phvul.006G114800,* regulated the expression of genes related to iron transporters and homeostasis in root and shoot [38]. Taken together, the evaluation of GWAS results from WGS imputed markers resulted in several plausible candidate genes that can be further evaluated with other methods.

## Discussion

This work reports the first MAGIC population in common bean, constructed from eight Mesoamerican breeding lines. MAGIC populations have been developed recently in several plant species, such as Arabidopsis [10], maize [39], rice [15], wheat [40], tomato [41] and *Vicia faba* [42]*,* among others. MAGIC populations are designed to capture more genetic variability and deliver more precise genetic mapping compared to commonly used bi-parental RIL populations. Utilization of breeding material produces results that are theoretically more easily transferable to breeding programs [15]. In this work, founder lines were selected to represent breeding variability in the CIAT Mesoamerican breeding program. These cover several market classes, an important aspect in common bean breeding, and furthermore, several important agronomic traits such as tolerance to drought, low fertility and aluminum, resistance to virus and high accumulation of Fe/Zn. Hence, these lines represent relevant allelic variation, applicable for several breeding efforts.

Recombinations are the basis of genetic mapping and generate the genetic variability for breeders’ selection. Due to several rounds of crossing, MAGIC populations offer increased recombinations and higher mapping precision than bi-parental populations. The MAGIC population presented here had on average 3.22 recombination events per chromosome. This value is expectedly higher than the averages observed for bi-parental RIL populations of common bean, ranging between 1.01 and 1.28 (DOR365xG19833, IJRxAFR298 and RCB593xINB841 populations, unpublished observations). In tomato, mean values of 2.49 and 1.46 recombination events were reported for MAGIC and bi-parental populations, respectively [41]. The rate of LD decay observed for this MAGIC population was 74 kbp at 0.19 ${r}_V^2$. This rate is faster than the 428 kbp reported for a natural population of cultivars/lines of the Mesoamerican genepool [43], Likewise, Blair et al. [44], describe a faster LD decay in an Andean population of common bean due to reduced population structure compared to a Mesoamerican population. Similar results have been reported for other MAGIC populations of winter-wheat [40] and cotton [45], and indicate that the consecutive rounds of mating may have broken linkage blocks, consequently reducing LD [45].

In this work, a high-density genetic map that incorporated multiple recombination events from eight founder lines using GBS SNP data was produced. This is the first map that incorporates such higher recombination frequency for common bean. It can be useful for improving the performance of some genotype imputation algorithms, incorporating recombination information with more precision [46]. In addition, the sigmoidal curves depicting recombination rates (Additional file 8) resembled those of previously published maps [44, 47]. However, the pericentromeric boundaries in the Mesoamerican founders’ genomes deviate from those defined in the reference genome of G19833 (Additional file 8). These results can be useful to study differences of local recombination events between the Mesoamerican and Andean gene pools. Furthermore, four founder lines were reported to bear interspecific introgressions from *P. coccineus* and *P. acutifolius* in chromosomes Pv07, Pv08, Pv09 and Pv11 [25]. Some of these regions display minor deviations on the proportional contribution (Fig. 4), but there is no clear signal of segregation distortion due to interspecific introgressions.

### Breeding applications of the MAGIC population

Breeding for drought tolerance has been a long-standing effort at CIAT’s and other common bean breeding programs. Yield has been used as a key selection criterion to advance the phenotypic responses under drought conditions.

One of the goals of the MAGIC population was to obtain lines with good agronomic performance and desirable QTL that are identified and tagged with markers to be used as resources for breeding programs. The average yield of the population was 763 and 1499 kg ha^− 1^ in 2013 and 2014 respectively, which is comparable to other populations phenotyped under drought conditions such as 1047 kg ha^− 1^ [8], 1030 kg ha^− 1^ [48], or 1173 kg ha^− 1^ [49]. Several RILs in the population showed superior performance compared to the founder lines. In this study, the top ten agronomically performing RILs were identified (Additional file 13). Some lines combine good yield performance with micro mineral levels. This is an important result because obtaining varieties with high yield and high accumulation of micronutrients has been a key challenge in the area of bean biofortification [6], due to the negative correlation between these two traits. Expectedly, the haplotypes of the major QTL that these lines carry do not completely explain performance, as many genetic factors that control these traits are not discovered. Yet, positive haplotypes of major QTL are enriched in the top lines. This suggests that MAS for major QTL and phenotypic selection should be combined to optimize genetic gain in selection.

Significant effort has been invested in the past in studies of physiology and genetics to obtain a deeper understanding of the agronomic responses under stress conditions beyond yield per se. The positive correlations between Yd and Yd components have previously been reported under drought conditions [48, 50–52]. In the same way, the observed negative correlations between Yd and phenology traits have been reported to be influenced by hot and dry growing conditions [53], revealing a strong dependence on the environmental factors for the relationship of these traits. Previous studies have used PHI as an effective selection criterion for identifying genotypes with improved drought tolerance [50]. Also, in this study, PHI had a positive phenotypic correlation with Yd. In general, these results show that superior yielding lines under drought stress are characterized by full commitment to reproductive growth, effective seed filling and resource translocation, leading to large seed and high PHI, as well as early maturity for drought avoidance.

QTL for agronomic traits have been widely studied in common bean using QTL mapping and GWAS studies [54]. DF is a key trait in the adaptation of common bean and QTL for DF and DPM have been previously reported in all chromosomes of the bean genome, except for Pv10. In this study, two major QTL in Pv01 for maturity were found, *DF1.1/DPM1.1* and *DF1.2/DPM1.2* in intervals 5–7 and 9–17 Mbp respectively. Other studies report QTL for DF in the same region also under drought stress, and showed that Mesoamerican genotypes contributed the additive allelic effect [22, 51, 53, 55, 56]. These data suggest there is an important locus on the start of chromosome Pv01 that controls maturity, next to the known *fin* locus at the lower arm of that chromosome in the interval 47–52 Mbp [57]. Likewise, other studies have reported QTL for DF and Yd located near to *DF3.1/DPM3.1* by linkage analysis and GWAS [57–59] and *DPM8.1* [56, 60].

QTL for seed weight have been previously reported in all chromosomes [54]. The main QTL found in this study for seed weight (*100SdW4.1*) represents new allelic variation for this oligogenic trait. The QTL hotspots (Pv01, Pv03, Pv06 and Pv08) identified in this work control both phenology and yield related traits, where the same alleles increase DF/DPM and decrease Yd and related traits. Hence, breeders may not be able to utilize these QTL for yield improvement, as primary consideration may have to be given to the maturity requirements for a certain target region and maturity is often negatively correlated with yield [53].

In this work, four PHI QTL were found on chromosomes Pv01, Pv02, Pv06 and Pv08 (Additional file 10). Mukeshimana et al. [51] reported two QTL under drought stress on Pv01 and one of these QTL was in the same region of *PHI1.1* (around 2 Mbp). Previous studies reported PHI QTL on chromosomes Pv06 and Pv08, at alternative chromosomal regions as identified here [53, 61]. *PHI6.1* could be useful in breeding in some genetic backgrounds, to avoid the negative allele from founder line MIB778, which was the lowest yielder in all trials. The main QTL found in this study for PHI (*PHI2.1*) has not been reported previously in other studies. *PHI2.1* appears to be independent from phenology traits and could be used for marker development, being associated with raising PHI values by 1.08%.

For seed Fe content, two main QTL located in Pv06 (*SdFe6.1* and *SdFe6.2*) are reported, contributed by MIB778, the founder with highest Fe and Zn accumulation. SdFe6.1 and SdFe6.2 have allelic effects of 2.1 and 2.7 ppm and could be employed in biofortification breeding. The allelic effects of the markers are moderate, also observed by Izquierdo et al. [62] and Ponce et al. [63]. This is according to expectation, as quantitative traits in elite breeding lines are not likely to vary in major effect loci, such as those reported for disease traits. Other QTL have been reported near to *SdFe6.1* and *SdFe6.2* in Andean populations [64, 65], a Mesoamerican population [66] and an inter-genepool population [67]. This data suggests that allelic variation at these loci exists in both Andean and Mesoamerican genepools. GWAS showed that positive alleles from MIB778 are mostly identical with the reference genome (Andean genotype G19833) alleles. Accordingly, this founder was reported to have a large Andean introgression on chromosome Pv06 in the same region [25]. The main QTL for seed Zn content (*SdZn8.1*) in this study was also based on the haplotype from MIB778. Previously reported QTL for Zn in Pv08 were located at an interval of ~ 8 Mbp from *SdZn8.1* [65, 66]. The founder line MIB778 was added to the MAGIC founders as a source for its high micromineral levels. It supplied the strongest QTL, which supports the semi-quantitative inheritance of this oligo genetic trait.

The responses to drought stress include morphological, physiological, biochemical and genetic mechanisms closely related, such as early flowering and maturity (escape mechanisms) [51], or the control of the root system architecture [68]. Some QTL related to this trait have been reported near the QTL hotspot identified in this study. A QTL controlling the number of root branches and root length diameter are located near to *DF1.1/DPM1.1* and *DPM8.1* respectively [69, 70]. It has been suggested that QTL affecting root traits in common beans are based on the constitutive expression of genes, and drought tolerance based on characteristics of the roots can be used in molecular breeding [71].

Previous studies on QTL for agronomic traits in common beans employed crosses between genetically contrasting parents, following the basic logic of QTL population development and mapping. This approach has been very successful in identifying and transferring disease resistance genes for, e.g. common bacterial blight (CBB) [72] or angular leaf spot resistance (ALS) [73]. It has also been used to analyze abiotic stress-related traits like drought tolerance [51, 56, 60] or tolerance to low levels of phosphorus [53]. However, the usefulness of this approach has been questioned for quantitative traits, because evaluating contrasting lines means to include alleles with undesirable agronomic effects for the investigated trait, which are likely to have been selected out of breeding populations. Strongly contrasting crosses are usually not used in breeding because it would result in much undesirable variation, particularly to regenerate commercial grain quality. For this reason, data from contrasting crosses is often not easily transferable to breeding applications. In contrast, genetic variation in a MAGIC population is analyzed in elite genetic backgrounds without detrimental exotic alleles; hence, data can be directly transferable to breeding populations.

### Comparison of genetic mapping methods: GWAS and QTL mapping

In this study, genotype-phenotype associations were simultaneously evaluated using linkage mapping with a method designed for eight-way MAGIC populations and using association mapping with the MLM approach. Using the QTL mapping strategy, the QTL *DF1.1*, *Yd1.1* and *DPM3.1* were identified in the two evaluations in 2013 and 2014. However, close-ups of these regions show that the significant LOD peaks do not overlap with each other, suggesting two different adjacent QTL that affect the trait independently in two seasons. Similarly, QTL mapping software IciMap [74] from the same author group was previously suggested to delimit QTL regions too much, leading to comparable problems of QTL proliferation in narrow regions, which does not appear biologically sensible [53]. In addition, the linkage strategy occasionally produced LOD peaks with very large significance scores that had no corresponding GWAS signal. Given that the interval QTL mapping software can utilize the known population and haplotype structure it should theoretically constitute a more powerful tool for genetic mapping, but the results presented here leave doubts if the data is completely reliable. On the other hand, GWAS results varied by applying different modifications of the GLM and MLM approaches (data not shown). Whereas major marker-trait associations are usually retained, significantly different results for intermediate and minor association regions can be obtained depending on the chosen analysis method, while no gold standard has been established in the literature for a correct analysis. For this reason, we reported reliable major QTL regions in this study that were supported by the significant signals produced by both the linkage and association strategies, providing enough evidence to delimit the associated regions, whereas only the GWAS results seem to be suitable to search for candidate genes.

### Value of WGS and imputed GWAS/QTL for candidate genes

Previous studies in common bean have identified candidate genes relying on either a priori candidates based on the published literature or comparing the functional annotation of gene models within a window, centered around significant SNPs markers e.g. [19, 22, 75–77]. The availability of WGS data for GWAS allows to largely delimit the list of potential candidate genes by removing those that do not show polymorphisms in the evaluated population. In this work, we performed GWAS using imputed WGS data from the founder lines. In principle, this could take in all polymorphisms in the population compared to the reference genome, including those that cause the phenotypic variation. This can lead to the accurate identification of candidate genes by tracing the polymorphism with the most significant association.

The strategy implemented in this study for gene mapping harbors some limitations. In the first place, the Andean reference genome of G19833 used here leaves out of the association analysis the unique Mesoamerican regions present in the founder lines. These regions would be included using a Mesoamerican reference, yet the reference genome of BAT93 still presented some contiguity and completeness issues, making it unsuitable to be used in this study. Furthermore, the founder lines likely contain introgressions from sister species, harboring unique coding regions that are not traceable and thus are left out. For example, *DPM8.1* is based on an allele from ALB213 where a *P. coccineus* introgression was reported previously [25]. Despite that, the mapping rates of the founders’ sequences were all close to 90%, which shows that most genomic regions that are common between both genepools have a low divergence, suitable for identifying and testing variants in the population. In addition, the polymorphism calling did not consider long structural variants (> 20 bp). Finally, we only evaluated modifications to the protein coding sequence of the reference genome to identify candidate genes; hence, any type of promoter mutations or non-annotated genes would be missed. This can be an important limitation and should be considered for further studies, because larger variants (insertions/deletions > 20 pb) can be found in promoter regions of the genes, causing functional changes such as those reported for the *GSE5* promoter in rice [78], *stiff1* promoter in maize [79] or PHYA3 promoter in common bean [80].

Following the approach of candidate gene identification using imputed WGS data, several candidate genes were identified for the evaluated agronomic quantitative traits. For the major phenology QTL on Pv01 and Pv08, many associated non-synonymous polymorphisms were found. Even though several plausible candidates are listed, no primary candidate stands out, which suggests that the genetic resolution, albeit quite large in this MAGIC population, is not enough to narrow down the candidate genes to a very small number. The strategy implemented in this study using resequencing of parental lines can be employed in other available RILs or multi-parental populations as a low-cost strategy for candidate gene identification. A similar strategy has been effective in the identification of QTLs for traits of variable genetic complexity in MAGIC populations of tomato and maize [39, 41] and the identification of candidate genes in rice [81].

All methods of candidate gene identification should be followed up by direct candidate gene validation. Some strategies of virus induced silencing or genetic transformation have been tested in common bean with a few successful reported cases [82–88]. It shows that candidate gene validation is still a complex task for the species and therefore not widely adopted up to date [24]. In that sense, EcoTILLING approaches could also be pursued to identify further functional alleles of a candidate gene in order to validate the gene function and identify further variability for breeding [83]. Currently only few WGS data sets have been published [25, 89, 90], but ongoing projects will make a much larger set of WGS data available in the near future that could be mined for allelic variation in genes of interest.

## Conclusions

This study presented the first common bean MAGIC population of the Mesoamerican gene pool. A genetic map comprising multiple recombination events between the founder lines was generated. To our knowledge, this map represents the largest and most dense genetic map available in common bean. The results presented here demonstrate that GWAS and haplotype-based interval mapping are successful tools in this population, identifying QTL for quantitative agronomic traits under drought conditions. Major QTL were identified to be controlling more than one trait, even in different seasons. This result is in line with the phenotypic correlations observed between some phenology and agronomic traits, suggesting there is extensive genetic correlation among them. Information on QTL can be used for molecular marker design for molecular breeding. Candidate genes for major QTL were identified using imputed WGS data from founder lines for GWAS. This method can be employed in RIL and MAGIC studies in common bean and other crops. Hence, this project provides data for applications in breeding and breeding tool development, especially for drought tolerance. This will support efforts to develop climate resilient germplasm, as well as information for basic research questions aiming to uncover the genetic basis of important agronomic traits.

## Methods

### Population development

Eight Mesoamerican common bean elite lines were selected from the breeding program at CIAT as founders of an 8-way MAGIC population: SXB412 (A), INB827 (B), ALB213 (C), SEN56 (D), SCR2 (E), MIB778 (F), SCR9 (G) and INB841 (H). These lines were selected based on genetic diversity (introgressions from *Phaseolus acutifolious*, *Phaseolus dumosus* and *Phaseolus coccineus*), phenotypic diversity for abiotic tolerance, micromineral concentration, disease resistance, and agronomic performance (Table 1).

The breeding scheme of the MAGIC population used in this study is shown in Additional File 1. In brief, the eight lines were crossed in four pairs to create F_1_ seed of SXB412 x INB827 (AxB), ALB213 x SEN56 (CxD), SCR2 x MIB778 (ExF) and SCR9 x INB841 (GxH). F_1_ plants were then intercrossed (AxB x CxD) and (ExF x GxH) to generate 323 “4-way” (ABCD) and 272 “4-way” (EFGH) F_1_ seed. These F_1_ plants were intercrossed once again (ABCD x EFGH) to generate 728 “8-way” (ABCDEFGH) F_1_ seed. 500 lines were randomly selected to advance to F_2_. Two plants per F_2_ family were selected to assure that the variability of segregation in the F_2_ would not be lost, and advanced to F_5_ through single seed descent, to obtain 996 RILs. DNA was collected from F_5_ individual plants. A first field trial was carried out with 636 RILs from the bulk harvested F_4.6_, in 2013. Trial sizes were limited by available field space; entries from the complete population were randomly selected. Individual F_5_ selections were advanced and 599 F_5.7_ RILs were phenotyped in the 2014 trial. Due to a communication error between programs, the two genotype sets were not identical, so that both trials share 437 RILs evaluated in the two seasons (Additional file 1).

### Field trial design and phenotyping

The MAGIC RILs and eight parents were planted at the International Center for Tropical Agriculture (CIAT) in Palmira, Colombia (with an altitude of 1000 m.a.s.l., latitude of 3° 32′ N and longitude of 76° 18′ W) in 2013 and 2014. The field experimental design for both trials was an alpha-lattice incomplete-block design with three replicates. Each genotype was laid out in two-row plots in 2013 and one-row plots in 2014 of 2.22 m^2^ each. Around 10% of the plots in 2013 were used for planting 5 different check lines evenly distributed across the field, and 7.5% of the plots in 2014 were used for founder lines randomly distributed across each replicate. Border plots surrounding the trial plots were planted in both trials. These trials were carried out in the dry season (precipitations: 96.3 mm in 2013 and 250 mm in 2014 throughout the crop cycle, average temperature: 24.7 °C in both seasons, see Additional file 2). Three irrigations were applied, the first three days before sowing and the two others at 10 and 21 days after sowing. Standard field practices were applied over the plant growing seasons across years including the application of fungicide seed treatment and foliar insecticides.

The number of days to flowering (DF) was measured from planting to the day when 50% of the plants in the plot had at least one open flower. Days to physiological maturity (DPM) was measured as the number of days from planting until 50% of plants had at least one pod losing its green pigmentation [50]. Yield (Yd, kg ha^− 1^) was obtained per plot and corrected for the percentage of moisture of the seed (seed moisture of 14%). Seed weight (100SdW, g 100 seeds^− 1^) was obtained from 100 seeds. These four traits were measured in both 2013 and 2014 trials. At the time of the harvest, 0.3 m^2^ per plot were collected separately to measure pod harvest index (PHI, %) defined as the ratio between seed weight to pod weight. PHI was measured only in 2013. The samples to evaluate iron and zinc concentration in the seed (SdFe and SdZn, ppm) were prepared according to the method described by Stangoulis and Sison [91] and quantified by X-ray fluorescence method using an Energy dispersive X-ray fluorescence (EDXRF) instrument X-Supreme 8000 (Oxford Instruments, UK) [92]. Micromineral concentration was evaluated in three replicates in 2014 and in a non-replicated trial in 2016. Additional information on description of phenotypic traits can be found in “Trait Dictionaries for Fieldbook Development” (www.cropontology.org).

### Phenotypic data analysis

The field map of the plots was used to assign row and column coordinates. The phenotypic data of each trial was analyzed by fitting a linear mixed model with random effects for rows and columns using the functions ‘SpATS’ and ‘PSANOVA’ [nseg = c (180,24) and nseg = c (36,54) for the trials in 2013 and 2014 respectively] of the R package SpATS (v1.0–9) [93]. To model the spatial variability in the field, this model contains a smooth bivariate surface composed of a parametric and a smoothing component. The parametric component includes the intercept, fixed linear trends along rows and columns and their linear interaction trend. The smoothing component models the deviation from the previous compound linear trend using one-dimensional and tensor product P-splines. The effect of each line on the phenotype was fitted as fixed and random to obtain the best linear unbiased estimators (BLUEs) and best linear unbiased predictors (BLUPs), respectively. The heritability was calculated using the function ‘getHeritability’ of SpATS, which uses the effective and nominal dimensions of the genotypic component calculated in the model [93]. Hence, the heritability was only obtained when the genotypic term was taken as random. BLUEs were used to calculate Pearson correlation coefficients among each trait-trial combination assessed in this study, and their significance was tested using a two-tailed *t-*test.

To select the top ten agronomically performing RILs, a weighted trait index (WTI) was constructed for each trial using BLUPs. This WTI included the traits Yd, DF and DPM with a weight of 40, 15 and 15% respectively. The remaining 30% was assigned to PHI in WTI of 2013 and SdFe in WTI of 2014. To construct the WTI, every trait was scaled using the *Z* transformation, assigning positive and negative scaled values to good and poor performing lines respectively (early maturity is interpreted as good performance). These values were combined using their corresponding weights for each trial separately. Then, the weighted averages for each trial were summed up and the top ten lines with highest values were selected.

### Genotyping

DNA extraction and sequencing of the founder lines are described in detail by Lobaton et al. [25]. In summary, the DNA was extracted from young leaves using liquid N_2_ and the urea buffer-based extraction miniprep protocol. The libraries were prepared using the Tru-Seq DNA PCR-Free library preparation kit, and the sequencing was performed by the HudsonAlpha Institute for Biotechnology, generating paired-end reads, yielding a raw sequencing depth ranging between 7x and 10x. The DNA extraction and genotyping by sequencing (GBS) of a subset of 629 MAGIC lines is described in detail by Perea et al. [94], following the same DNA extraction protocol described above. The DNA was sent to the Cornell sequencing facility for the GBS library preparation using the restriction enzyme *ApeKI* [95]. Each plate of 96-wells was sequenced in two lanes of an Illumina HiSeq platform using single-end reads. On average, each sample had a raw sequencing depth of 0.4x.

The mapping and variant calling processes for the founder lines is described in detail by Lobaton et al. [25]. Multi-allelic sites in the identified variants were split into bi-allelic sites using the module ‘norm’ with the options -m and -f from bcftools (v1.8) [96], yielding a total of 6,284,436 bi-allelic variants from the founders’ sequencing data (Additional file 4). The GBS reads were demultiplexed using NGSEP (v3.1.2) [97]. Adapters and low-quality bases from the raw sequencing data were trimmed using Trimmomatic (v0.36) [98], and the processed reads were aligned to the reference genome of *P. vulgaris* accession G19833 v2.1 [46]. using Bowtie2 (v2.2.30) [99] with default parameters. The variant calling process was performed using NGSEP following recommended parameters for GBS data [94]. The list of variants identified previously in the founder lines was used as the variants to be genotyped in the MAGIC lines. The resulting genotype matrix was filtered for variants with a genotype quality above 40, MAF above 0.05, and at least 260 individuals genotyped per site. The final genotype matrix from GBS data contained 20,615 variants with ~ 25% missing genotype calls in the whole matrix (Additional files 4 and 5). The GBS matrix was used to assess the population structure in the MAGIC lines by performing a principal component analysis (PCA) using GAPIT (v3.0) [100], and constructing an unrooted neighbor-joining (NJ) tree using SplitsTree (v4.14.16) [101]. The GBS matrix was also used to calculate pairwise measures of LD in sliding windows of 100 markers for each chromosome. The LD measures were corrected for kinship relationships in the population (${r}_V^2$) as implemented in the R package LDcorSV (v1.3.2) [102]. The LD decay was estimated regressing the pairwise ${r}_V^2$ values on the physical distance of their markers using the locally estimated scatterplot smoothing implemented in the R function ‘loess’ (v3.6.3), with a span value of 0.5.

To quantify the proportional contribution of genomic information from the founders’ genomes to the MAGIC lines, the parental haplotype blocks in the population were estimated using the method proposed for haplotype prediction in an *Arabidopsis thaliana* MAGIC population [10] (http://mtweb.cs.ucl.ac.uk/mus/www/19genomes/magic.html). This was performed using the whole set of founders and GBS markers, masking out indel variants and repetitive regions in the reference genome [25]. This method infers the breakpoints in the haplotypes by a dynamic programming algorithm, akin to the Viterbi path from a hidden Markov model (HMM). To construct a genetic linkage map, the inferred haplotype blocks and the GBS matrix were used to calculate genetic distances among markers using the Kosambi mapping function implemented in the integrated genetic analysis software for multi-parental pure-line populations (GAPL v1.2) [103]. The GBS marker set in this matrix was reduced by a binning procedure based on their recombination frequency as implemented in GAPL, generating a subset of 5738 non-redundant markers (Additional file 4). To impute the founders’ WGS variants in the RILs, the founders’ markers and the GBS markers were merged into a single matrix of 6,284,436 variants and 637 individuals. The missing data in this matrix was imputed with Beagle (v5.0) [46], providing the genetic linkage map (5738 non-redundant markers) and setting the effective population size to 8. The final imputed matrix was filtered for variants with MAF below 0.05, producing a matrix of 1,972,528 markers with genotype calls for all 637 samples (Additional files 4 and 5) that was used thereafter for the GWAS analyses.

### QTL analysis and GWAS

QTL analysis was conducted using the genetic map described above and the genotypic BLUPs from the trials separately. Detection of QTL and estimation of the genetic parameters for each trait evaluated were performed using the composite interval mapping with the procedure for additive effects (ICIM-ADD) of the software GAPL (v1.2) [103], employing the forward and backward regression model, with a 5 cM window size and a 0.5 cM sliding window. A significant QTL was declared if the logarithm of odds (LOD) was greater than the significance threshold of 6.26, which was obtained by performing a permutation test 1000 times with a *p* value of 0.05 to minimize the experimental type-I error rate. To identify the best allele for each QTL, a Tukey’s multiple comparison test (*α* = 0.05) was performed using the founders’ haplotypes, assessing the effect of each haplotype separately.

Based on the results from the population structure assessment, different modifications to the general linear model (GLM) and the mixed linear model (MLM) approaches were tested for GWAS. This test included variable number of principal components as covariates, different methods to calculate the genetic relatedness (kinship) matrix, and different model implementations in the software packages Tassel (v5.2.44), GAPIT (v3.0) [100] and GENESIS (v2.8.1) [104] (data not shown). The selection criteria for the tested models was based on the calculation of the mean squared difference (MSD) between the observed and expected *p* values [105] The model with the lowest MSD was obtained with the R package GENESIS (https://github.com/UW-GAC/GENESIS). This model accounts for population structure using the top five principal components (described previously) as fixed effects. It also accounts for random polygenic effects with a kinship matrix as variance-covariance structure, calculated using the EMMA algorithm implemented in GAPIT (v3.0) [100]. Significant associations with the trait of interest were declared when the *p* value was equal to or smaller than the Bonferroni threshold calculated with the GBS markers (2.42 × 10^− 6^ with 20,615 markers). The association and linkage analyses were performed using a subset of 444 lines for the 2013 trial and 602 lines for the 2014 and 2016 trials, for which genotypic and phenotypic data was available.

### Candidate gene identification

To detect putative candidate genes and candidate polymorphisms affecting the phenotypic variation, the major QTL regions identified by both QTL analysis and GWAS were selected. This search was restricted to the significant variants that had a high or moderate effect in the coding regions of the reference genome as defined by the annotation using SnpEff (v4.3) [106]. In addition, the gene expression data reported by O’Rourke et al. [107] and Phytozome (v12.1.6) (https://phytozome.jgi.doe.gov) was used to check if the selected genes had relevant expression levels in the tissue of interest. A gene was declared a candidate if its gene ontology description in Phytozome (v12.1.6) included a function related to the trait evaluated.

## Supplementary Information

**Additional file 1.** (a) Crossing and selection scheme of the common bean MAGIC population. (b) Venn diagram showing the number of RILs used for genotyping and phenotyping in each trial.

**Additional file 2.** Precipitation, maximum and minimum temperatures during trials at Palmira, Colombia.

**Additional file 3.** Phenotypic variability, least significant difference (LSD) and broad-sense heritability (H^2^) for best linear unbiased predictors (BLUPs) of the evaluated traits in the trials of 2013, 2014 and 2016 of the MAGIC population.

**Additional file 4.** Distribution of markers per chromosome obtained from WGS of the eight founder lines. Markers from GBS of the whole population and the resulting thinned markers used to construct the genetic map for QTL analysis are listed.

**Additional file 5.** Heat map of the density of markers called from WGS and GBS along the eleven chromosomes of the *P. vulgaris* reference genome. Each color band represents a region of 250 kbp. The inner black lines represent the boundaries of the pericentromeric regions as defined by Schmutz et al. [47].

**Additional file 6.** Pedigree tree for six of the eight founder lines of the MAGIC population. The founders are highlighted in dark gray at the lower tips of the tree. Exact pedigrees of INB lines are not currently available. This tree was generated using Helium (v1.18.03.15).

**Additional file 7.** Haplotype composition of the MAGIC lines. The parental source, the length and genomic construction of each haplotype in each MAGIC line is shown, using the method proposed by Kover et al. [10].

**Additional file 8.** Comparison between the physical location and the recombination frequency (genetic map position) based on thinned GBS markers (5.738 markers). The dashed horizontal lines represent the boundaries of the pericentromeric regions as defined by Schmutz et al. [47].

**Additional file 9.** Pattern of linkage disequilibrium (LD) decay calculated genome-wide (black-dashed line) and for each chromosome separately (colored lines) in a MAGIC population of common beans. The pairwise measures of LD were calculated in sliding windows of 100 markers and corrected for kinship relationships in the population (${r}_V^2$). Each line corresponds to a locally estimated scatterplot smoothing (LOESS) regression on the LD measures.

**Additional file 10.** Significant markers identified in genome wide association studies, genetic and physical position, *p* value, allele frequency, phenotypic effect and founder genotypes associated with 9 traits in the MAGIC population evaluated in 2013, 2014 and 2016. Favorable alleles are colored in green.

**Additional file 11.** Details of QTL identified by interval mapping, genetic and physical position, LOD, phenotypic variation explained, and founders’ allelic effects mapped for 7 traits in the MAGIC population evaluated in 2013, 2014 and 2016.

**Additional file 12.** Manhattan, quantile-quantile and LOD plots of the association and linkage mapping for each of the evaluated traits. The Bonferroni correction threshold (*p* = 0.05) using the WGS (1,972,528) and the GBS (20,615) markers are depicted as red and green horizontal dashed lines, respectively, in the Manhattan plot. The significance threshold for the QTL mapping analysis is depicted as the blue dashed line in the LOD plot.

**Additional file 13.** Phenotypic performance of top 10 RILs of the common bean MAGIC population and their genotypes at 12 major QTL. Phenotypic values for each of the top ten MAGIC RIL lines are shown for each trial, color coded from desirable (green) to undesirable (red) values. For each RIL line founder haplotypes for the top 12 QTL are shown together with the haplotype effects color coded from desirable (blue) to undesirable (red) QTL effects. The data mean phenotype for each parental haplotype in the main QTL and maximum and minimum trait values or haplotype effects are shown below. Letters indicate significant differences using Tukey test (α = 0.05).

**Additional file 14.** Haplotype composition per chromosome of the top 10 RILs of the common bean MAGIC population.

**Additional file 15.** Significant polymorphisms in major QTL identified by both methods MLM GWAS and QTL mapping based on haplotypes that affect protein coding regions of candidate genes with moderate or high effect as defined by SnpEff (v4.3). Gene expression data added from O’Rourke et al. [105] and Phytozome.org (v12.1.6).

## Data Availability

The raw sequencing data used in this study is available at the NCBI sequence read archive (SRA) database with the bioproject accession numbers PRJNA294602 and PRJNA289910 as published by Perea et al. [82] and Lobaton et al. [25]. The variants for the eight founder lines used in this study are available for downloading at dryad (10.5061/dryad.46pk7) and for browsing at the European Variation Archive database of the European Bioinformatics Institute with the accession number PRJEB18671 and biosample accession numbers SAMN07312849, SAMN07312850, SAMN07312851, SAMN07312852, SAMN07312853, SAMN07312854, SAMN07312855 and SAMN07312856. The GBS matrix and the genetic map, as well as raw and modeled phenotypic data used in this study are available for download at Harvard Dataverse (10.7910/DVN/JR4X4C).

## Abbreviations

MAGIC: Multiparent advanced generation intercross
GBS: Genotyping by sequencing
WGS: Whole genome sequencing
QTL: Quantitative trait loci
GWAS: Genome-wide association study
MNM: Micronutrient malnutrition
MAS: Marker-assisted selection
LD: Linkage disequilibrium
RIL: Recombinant inbred line
Yd: Yield
DF: Days to flowering
DPM: Days to physiological maturity
100SdW: Seed weight
PHI: Pod harvest index
SdFe: Iron accumulation in seed
SdZn: Zinc accumulation in seed
MLM: Mixed linear model
GxE: Genotype x environment
WTI: Weighted trait index
CBB: Common bacterial blight
ALS: Angular leaf spot
EDXRF: Energy dispersive X-ray fluorescence
BLUEs: Best linear unbiased estimators
BLUPs: Best linear unbiased predictors
PCA: Principal component analysis
NJ: Neighbor-joining
HMM: Hidden Markov model
LOD: Logarithm of odds
GLM: General linear model
