# Supplementary material for: Genetic mapping for agronomic traits in a MAGIC population of common bean (Phaseolus vulgaris L.) under drought conditions
Source: BMC Genomics. 2020 Nov 16;21:799. doi: 10.1186/s12864-020-07213-6 (PMC7670608; doi:10.1186/s12864-020-07213-6)
Supplement: Supplementary file 1 — Additional file 1. (a) Crossing and selection scheme of the common bean MAGIC population. (b) Venn diagram showing the number of RILs used for genotyping and phenotyping in each trial. [file 12864_2020_7213_MOESM1_ESM.pdf]

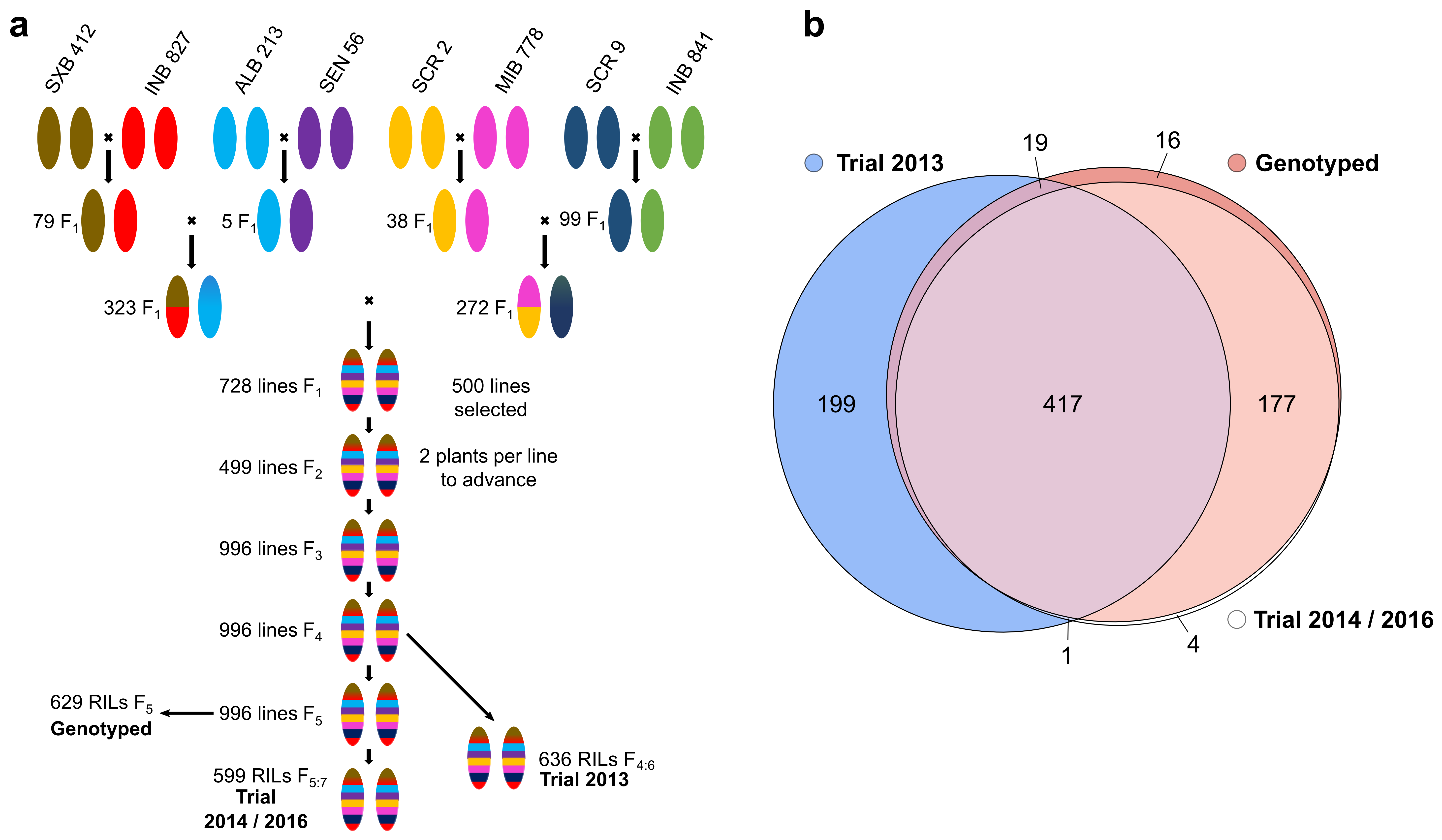

Additional file 1. a. Crossing and selection scheme of the common bean MAGIC population. **b.** Venn diagram showing number of RILs used for genotyping and phenotyping in each trial.
