# Supplementary material for: Genetic mapping for agronomic traits in a MAGIC population of common bean (Phaseolus vulgaris L.) under drought conditions
Source: BMC Genomics. 2020 Nov 16;21:799. doi: 10.1186/s12864-020-07213-6 (PMC7670608; doi:10.1186/s12864-020-07213-6)
Supplement: Supplementary file 2 — Additional file 2. Precipitation, maximum and minimum temperatures during trials at Palmira, Colombia. [file 12864_2020_7213_MOESM2_ESM.pdf]

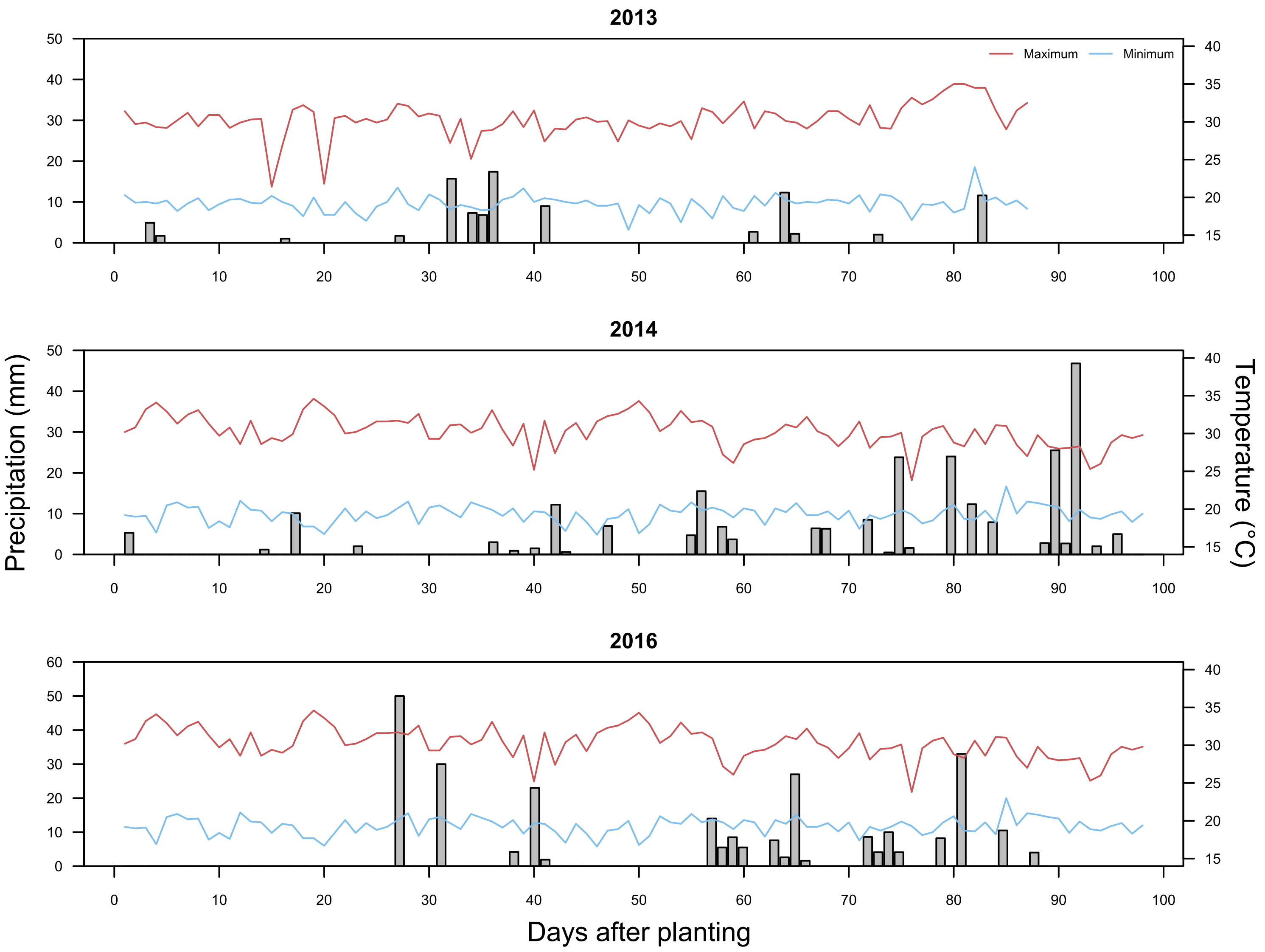

Additional file 2. Precipitation, maximum and minimum temperatures during trials at Palmira, Colombia.
