# Supplementary material for: Genetic mapping for agronomic traits in a MAGIC population of common bean (Phaseolus vulgaris L.) under drought conditions
Source: BMC Genomics. 2020 Nov 16;21:799. doi: 10.1186/s12864-020-07213-6 (PMC7670608; doi:10.1186/s12864-020-07213-6)
Supplement: Supplementary file 5 — Additional file 5. Heat map of the density of markers called from WGS and GBS along the eleven chromosomes of the P. vulgaris reference genome. Each color band represents a region of 250 kbp. The inner black lines represent the boundaries of the pericentromeric regions as defined by Schmutz et al. [47]. [file 12864_2020_7213_MOESM5_ESM.pdf]

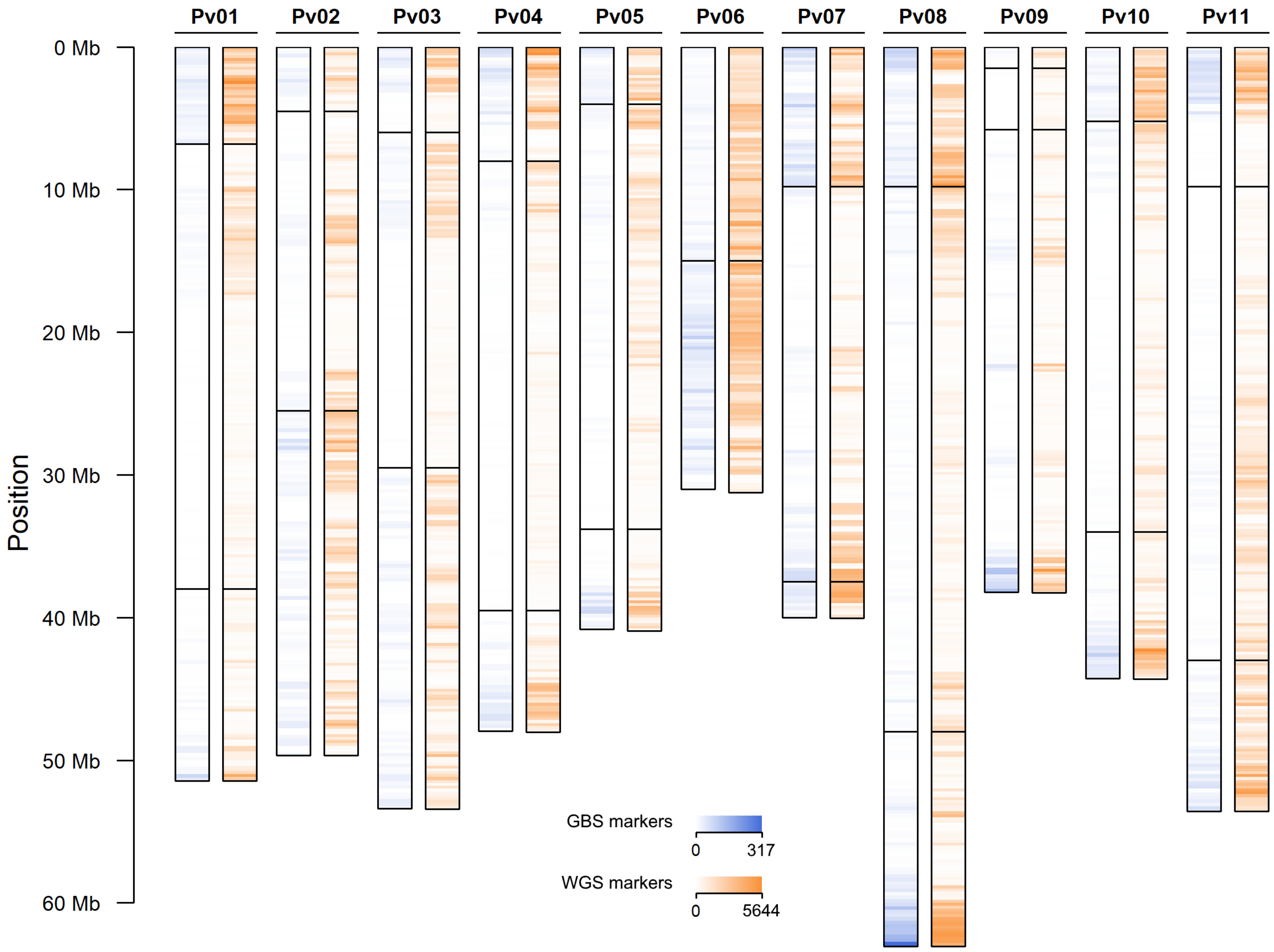

Additional file 5. Heat map for the density of markers called from WGS and GBS along the eleven chromosomes of the *P. vulgaris* reference genome. Each color band represents a region of 250 kbp. The inner black lines represent the boundaries of the pericentromeric regions as defined by Schmutz et al. (2014).
