# Supplementary material for: Genetic mapping for agronomic traits in a MAGIC population of common bean (Phaseolus vulgaris L.) under drought conditions
Source: BMC Genomics. 2020 Nov 16;21:799. doi: 10.1186/s12864-020-07213-6 (PMC7670608; doi:10.1186/s12864-020-07213-6)
Supplement: Supplementary file 6 — Additional file 6. Pedigree tree for six of the eight founder lines of the MAGIC population. The founders are highlighted in dark gray at the lower tips of the tree. Exact pedigrees of INB lines are not currently available. This tree was generated using Helium (v1.18.03.15). [file 12864_2020_7213_MOESM6_ESM.pdf]

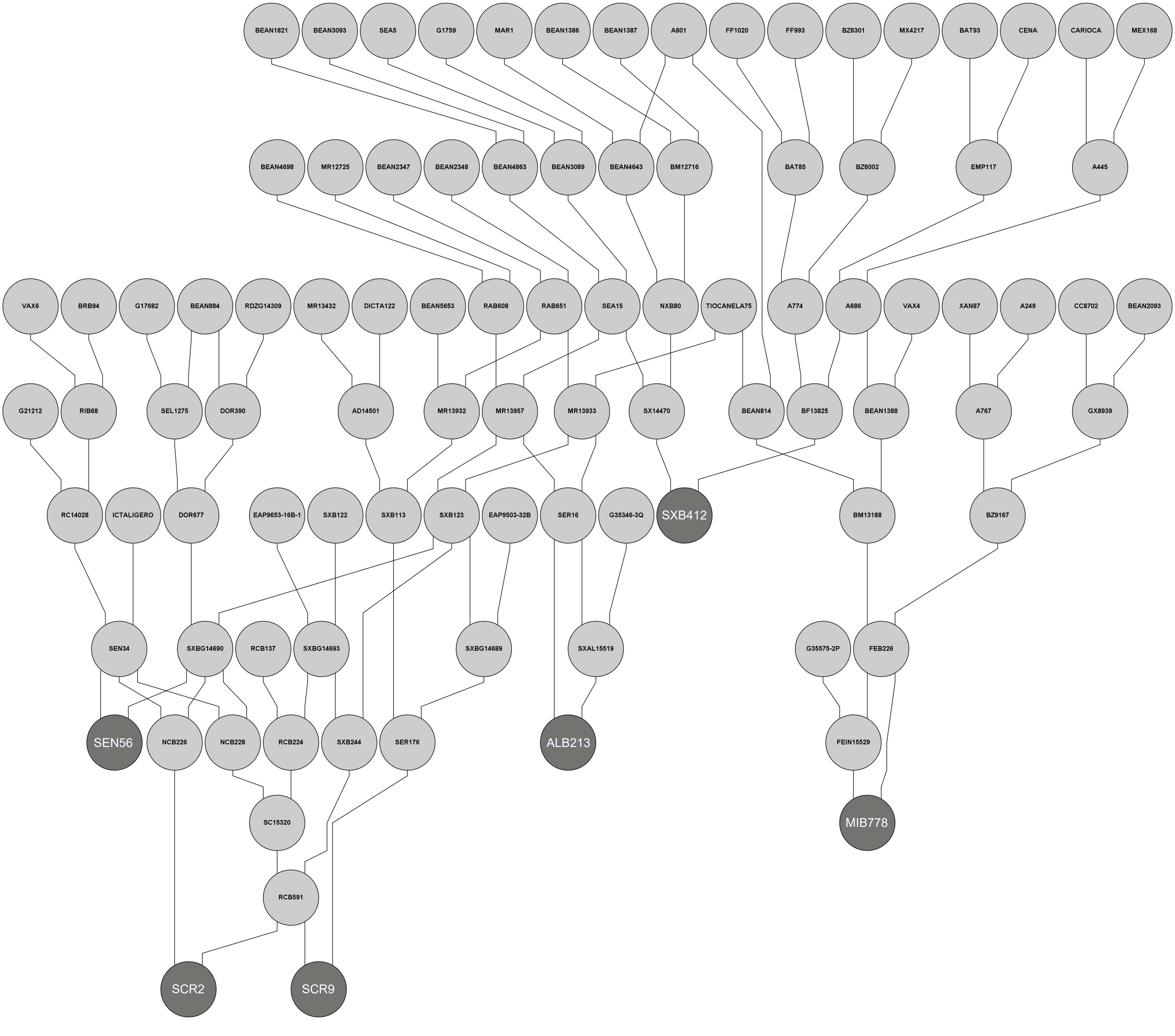

Additional file 6. Pedigree tree for six of the eight founder lines of the MAGIC population. The founders are highlighted in dark gray at the lower tips of the tree. Exact pedigrees of INB lines are not currently available. This tree was generated using Helium (v1.18.03.15) (Shaw et al. 2014).
