# Supplementary material for: Genetic mapping for agronomic traits in a MAGIC population of common bean (Phaseolus vulgaris L.) under drought conditions
Source: BMC Genomics. 2020 Nov 16;21:799. doi: 10.1186/s12864-020-07213-6 (PMC7670608; doi:10.1186/s12864-020-07213-6)
Supplement: Supplementary file 8 — Additional file 8. Comparison between the physical location and the recombination frequency (genetic map position) based on thinned GBS markers (5.738 markers). The dashed horizontal lines represent the boundaries of the pericentromeric regions as defined by Schmutz et al. [47]. [file 12864_2020_7213_MOESM8_ESM.pdf]

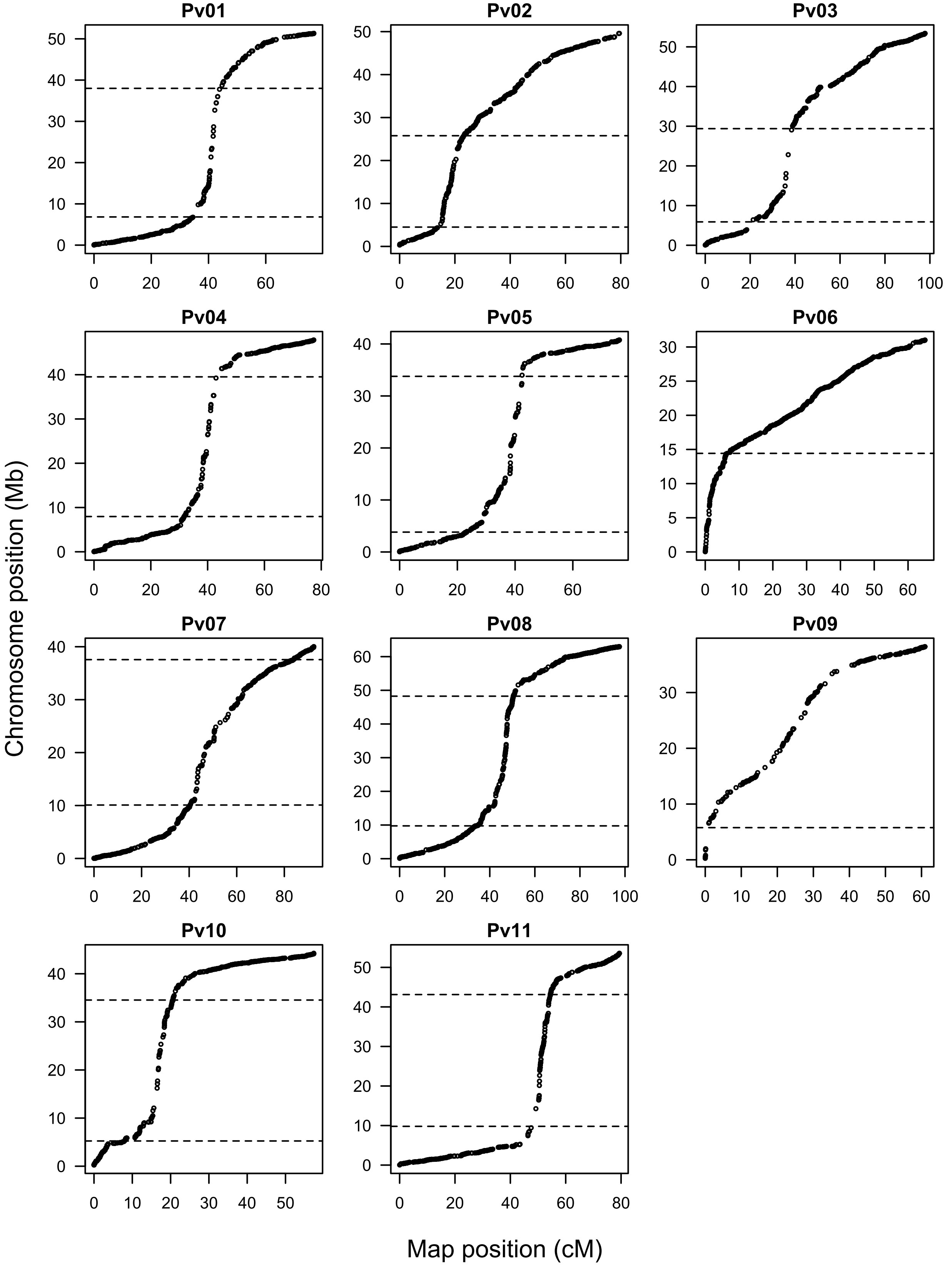

Additional file 8. Comparison between the physical location and the recombination frequency (genetic map position) based on thinned GBS markers (5.738 markers). The dashed horizontal lines represent the boundaries for the pericentromeric regions as defined by Schmutz et al. (2014).
