# Supplementary material for: Genetic mapping for agronomic traits in a MAGIC population of common bean (Phaseolus vulgaris L.) under drought conditions
Source: BMC Genomics. 2020 Nov 16;21:799. doi: 10.1186/s12864-020-07213-6 (PMC7670608; doi:10.1186/s12864-020-07213-6)
Supplement: Supplementary file 9 — Additional file 9. Pattern of linkage disequilibrium (LD) decay calculated genome-wide (black-dashed line) and for each chromosome separately (colored lines) in a MAGIC population of common beans. The pairwise measures of LD were calculated in sliding windows of 100 markers and corrected for kinship relationships in the population (${r}_V^2$rV2). Each line corresponds to a locally estimated scatterplot smoothing (LOESS) regression on the LD measures. [file 12864_2020_7213_MOESM9_ESM.pdf]

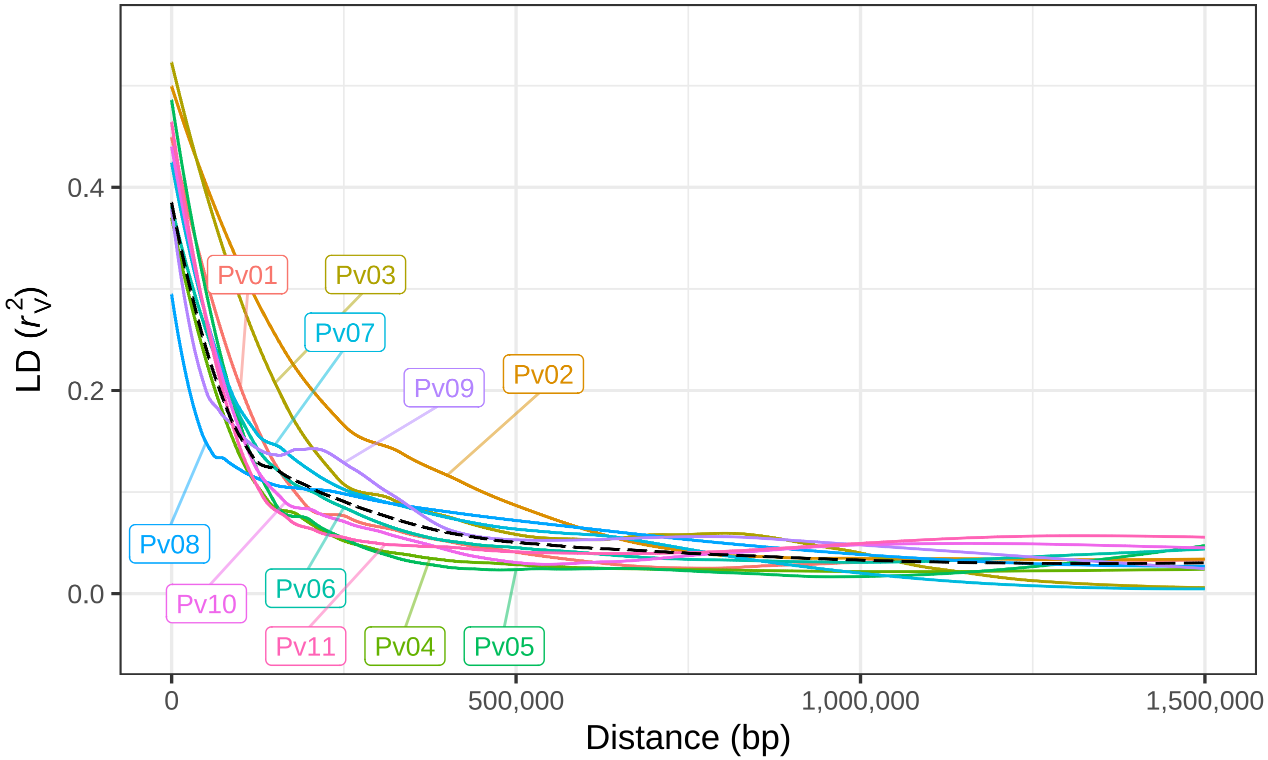

Additional file 9. Pattern of linkage disequilibrium (LD) decay calculated genome−wide (black−dashed line) and for each chromosome separately (colored lines) in a MAGIC population of common bean. The pairwise measures of LD were calculated in sliding windows of 100 markers and corrected for kinship relationships in the population (r_V^2). Each line corresponds to a locally estimated scatterplot smoothing (LOESS) regression on the LD measures.
