# Supplementary material for: Genetic mapping for agronomic traits in a MAGIC population of common bean (Phaseolus vulgaris L.) under drought conditions
Source: BMC Genomics. 2020 Nov 16;21:799. doi: 10.1186/s12864-020-07213-6 (PMC7670608; doi:10.1186/s12864-020-07213-6)
Supplement: Supplementary file 12 — Additional file 12. Manhattan, quantile-quantile and LOD plots of the association and linkage mapping for each of the evaluated traits. The Bonferroni correction threshold (p = 0.05) using the WGS (1,972,528) and the GBS (20,615) markers are depicted as red and green horizontal dashed lines, respectively, in the Manhattan plot. The significance threshold for the QTL mapping analysis is depicted as the blue dashed line in the LOD plot. [file 12864_2020_7213_MOESM12_ESM.pdf]

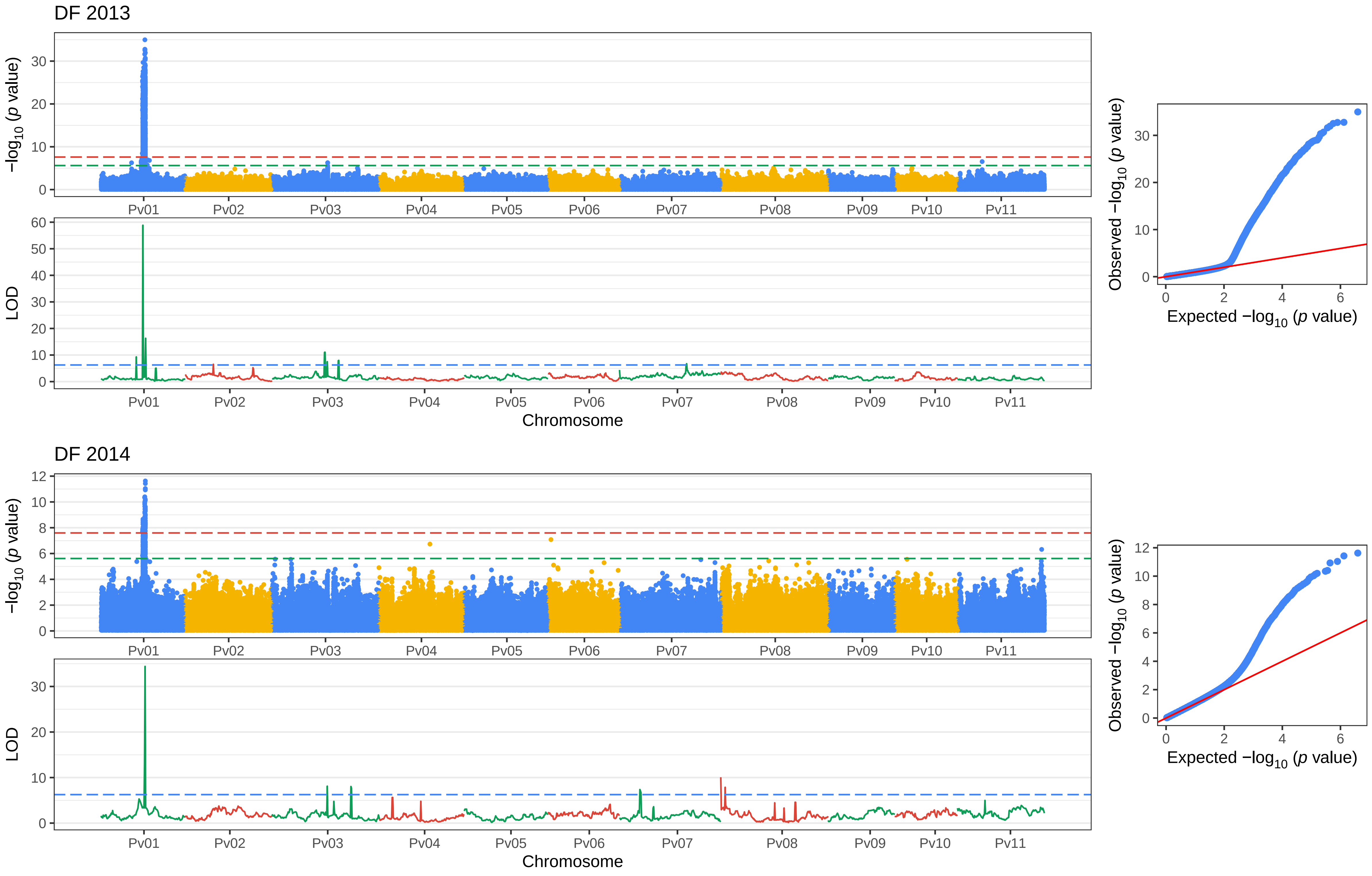


**Additional file 12.** Manhattan, quantile−quantile and LOD plots of the association and linkage mapping for each of the evaluated traits. The Bonferroni correction threshold ($p$ = 0.05) using the WGS (1,972,528) and the GBS (20,615) markers are depicted as red and green horizontal dashed lines, respectively, in the Manhattan plot. The significance threshold for the QTL mapping analysis is depicted as a blue dashed line in the LOD plot.

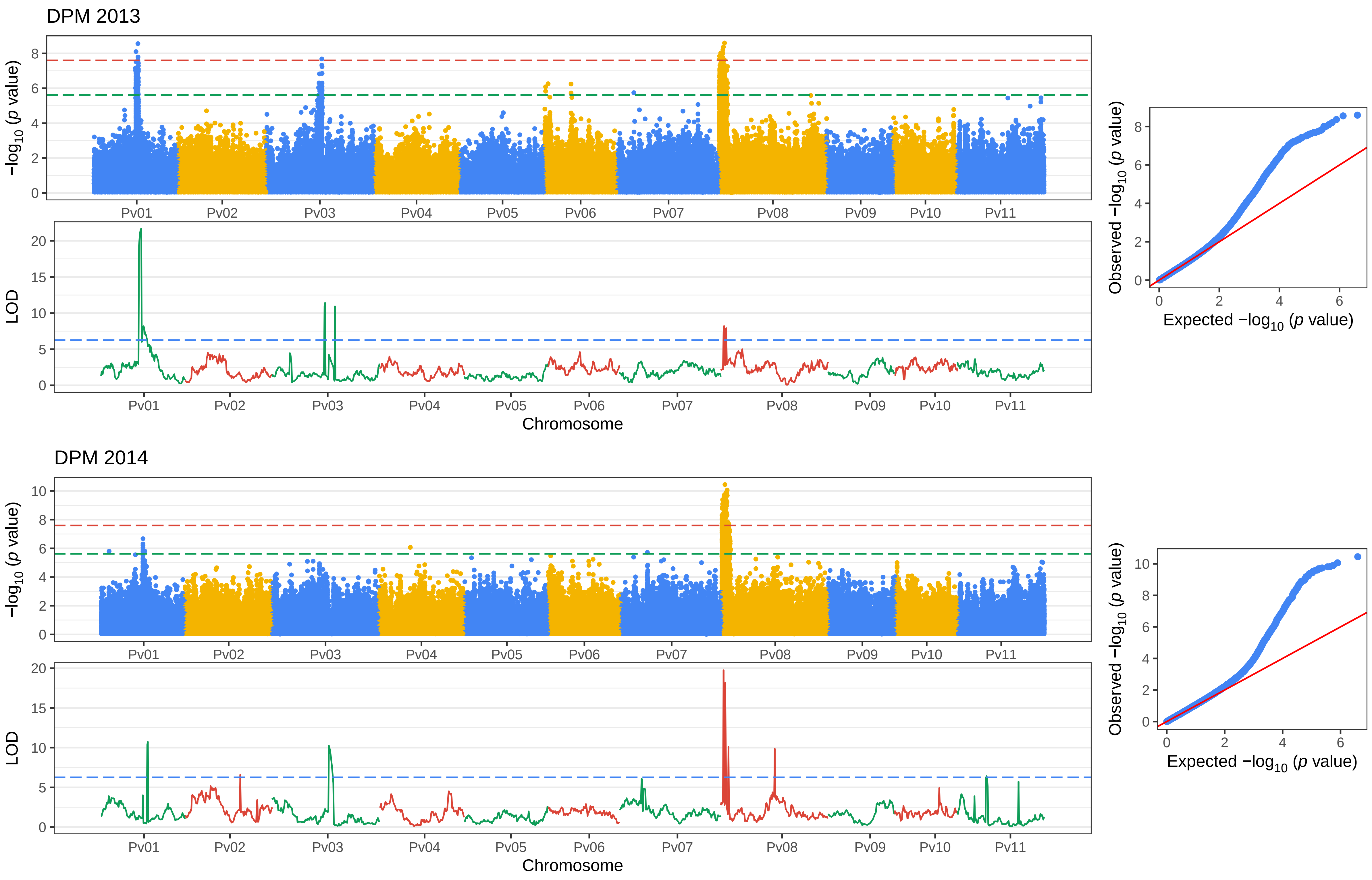


**Additional file 12.** Manhattan, quantile−quantile and LOD plots of the association and linkage mapping for each of the evaluated traits. The Bonferroni correction threshold ($p$ = 0.05) using the WGS (1,972,528) and the GBS (20,615) markers are depicted as red and green horizontal dashed lines, respectively, in the Manhattan plot. The significance threshold for the QTL mapping analysis is depicted as a blue dashed line in the LOD plot.

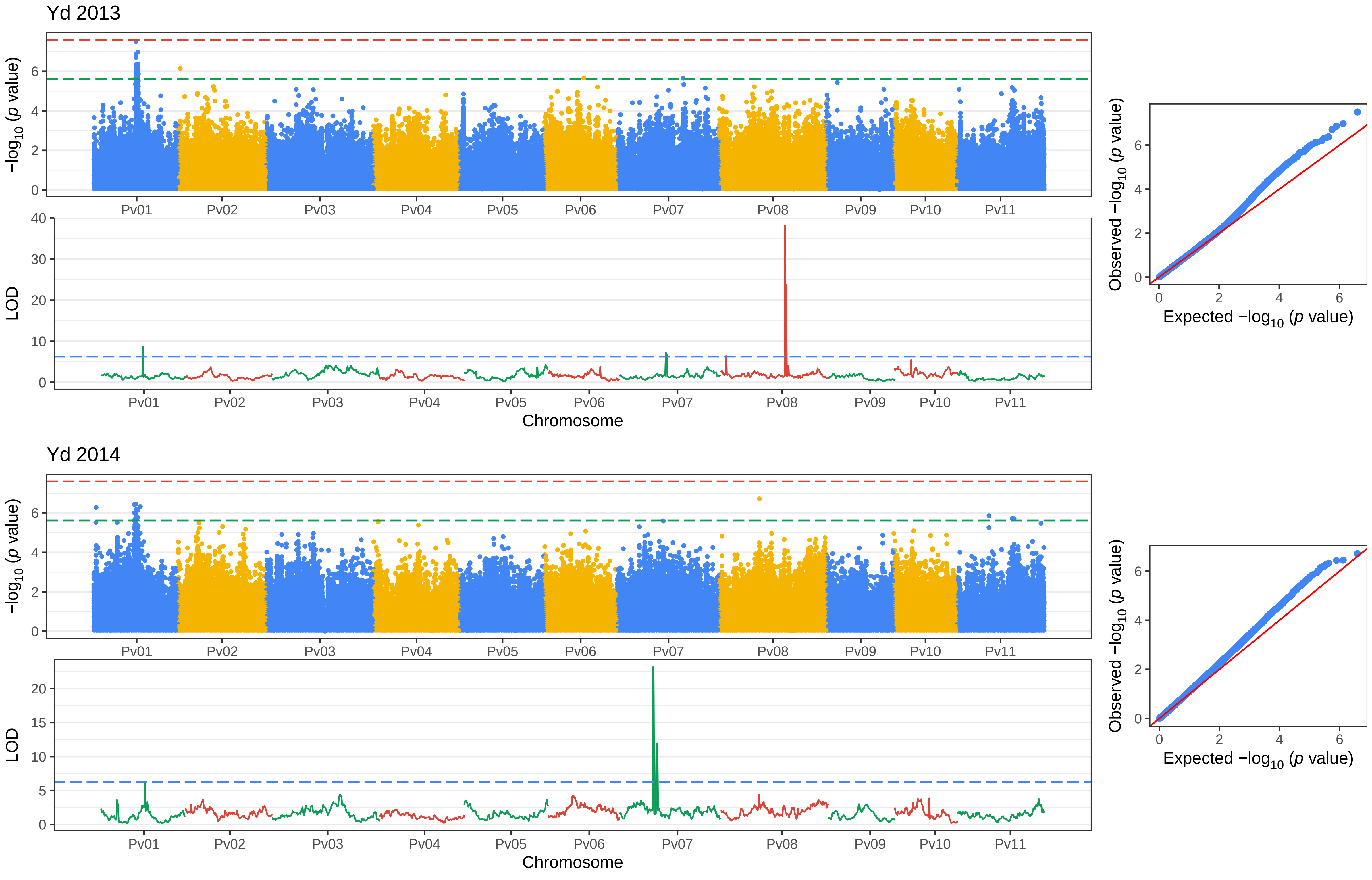


**Additional file 12.** Manhattan, quantile−quantile and LOD plots of the association and linkage mapping for each of the evaluated traits. The Bonferroni correction threshold ($p$ = 0.05) using the WGS (1,972,528) and the GBS (20,615) markers are depicted as red and green horizontal dashed lines, respectively, in the Manhattan plot. The significance threshold for the QTL mapping analysis is depicted as a blue dashed line in the LOD plot.

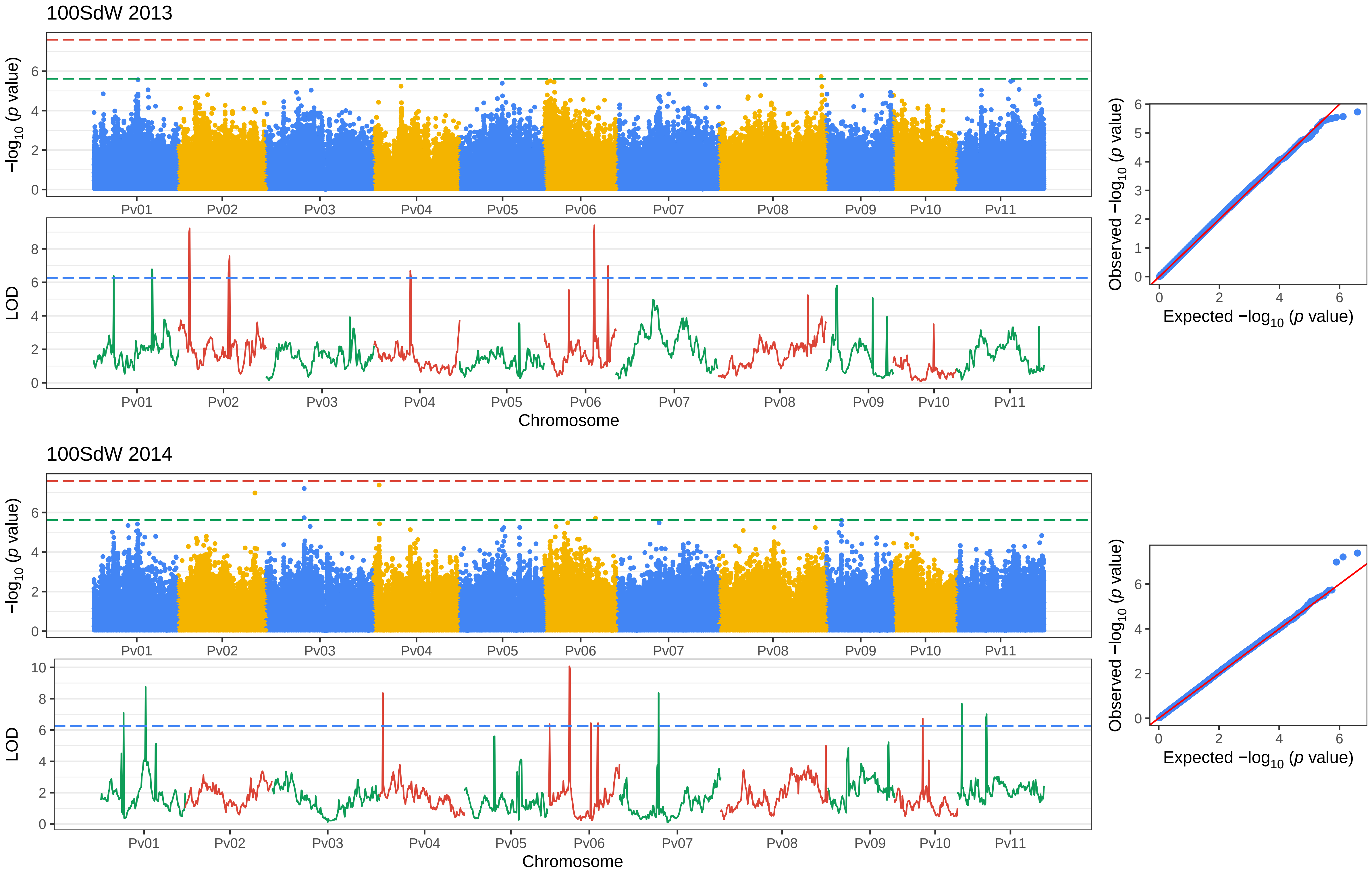


**Additional file 12.** Manhattan, quantile−quantile and LOD plots of the association and linkage mapping for each of the evaluated traits. The Bonferroni correction threshold ($p$ = 0.05) using the WGS (1,972,528) and the GBS (20,615) markers are depicted as red and green horizontal dashed lines, respectively, in the Manhattan plot. The significance threshold for the QTL mapping analysis is depicted as a blue dashed line in the LOD plot.

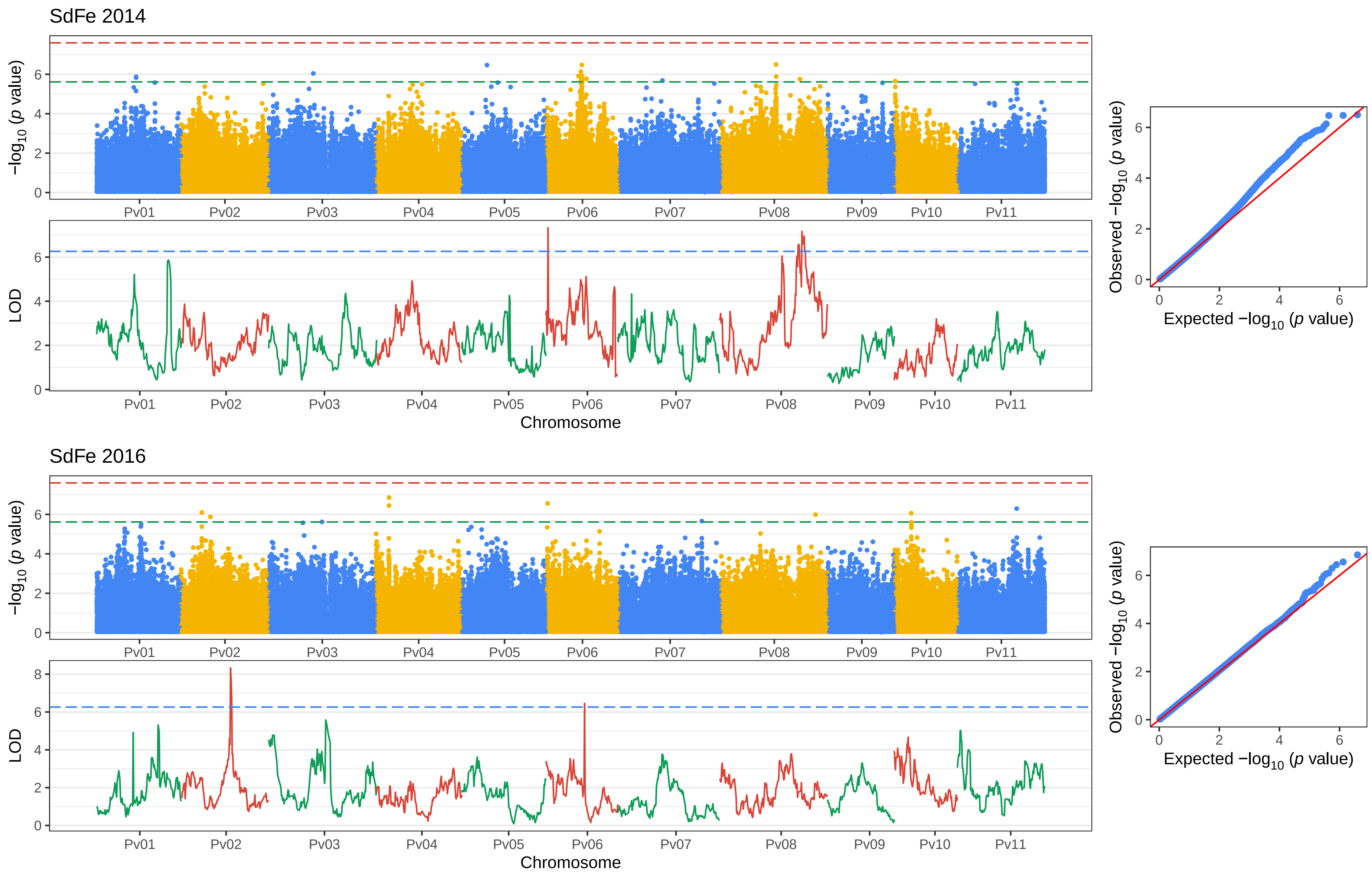


**Additional file 12.** Manhattan, quantile−quantile and LOD plots of the association and linkage mapping for each of the evaluated traits. The Bonferroni correction threshold ($p$ = 0.05) using the WGS (1,972,528) and the GBS (20,615) markers are depicted as red and green horizontal dashed lines, respectively, in the Manhattan plot. The significance threshold for the QTL mapping analysis is depicted as a blue dashed line in the LOD plot.

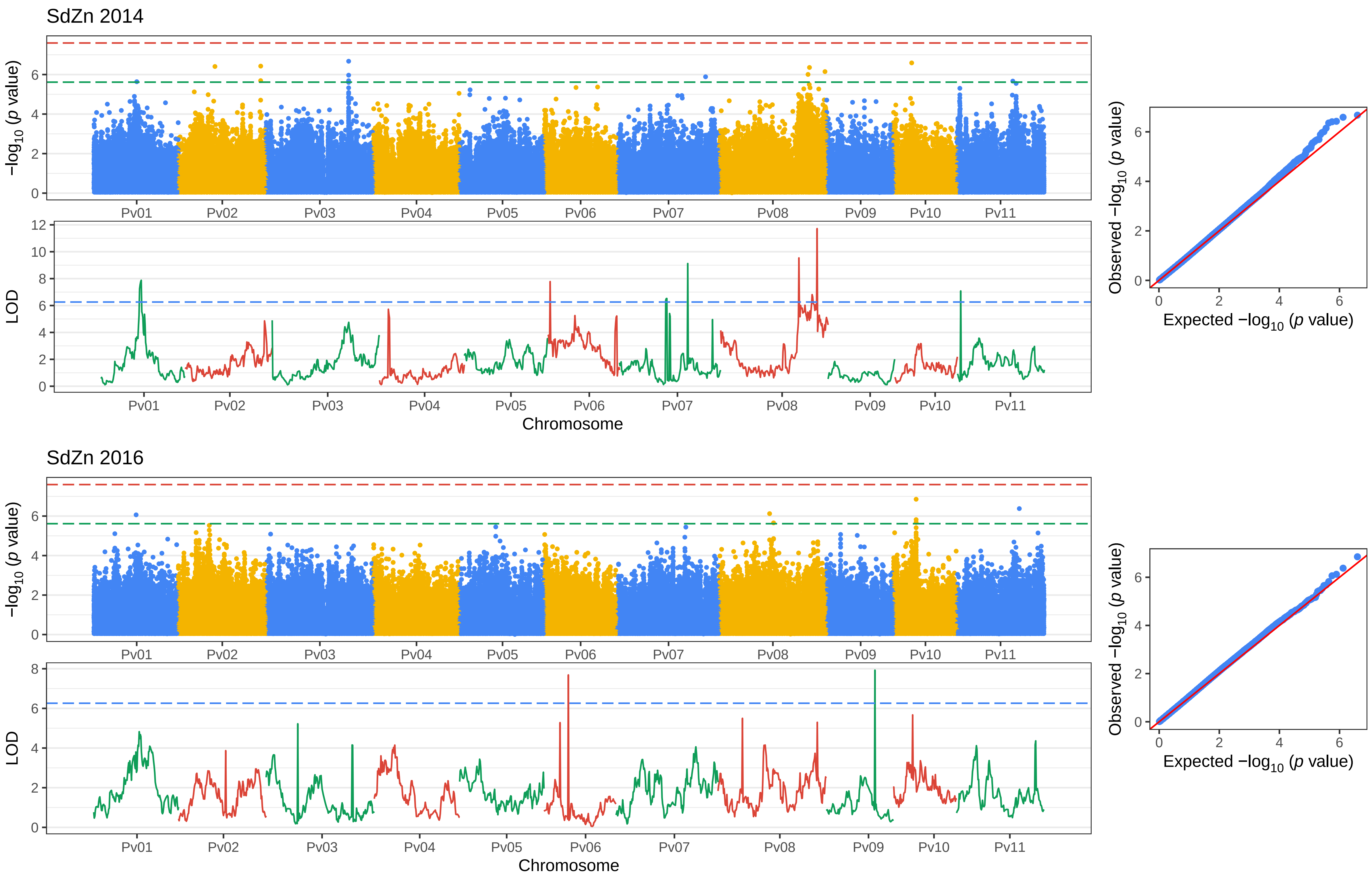


**Additional file 12.** Manhattan, quantile−quantile and LOD plots of the association and linkage mapping for each of the evaluated traits. The Bonferroni correction threshold ($p$ = 0.05) using the WGS (1,972,528) and the GBS (20,615) markers are depicted as red and green horizontal dashed lines, respectively, in the Manhattan plot. The significance threshold for the QTL mapping analysis is depicted as a blue dashed line in the LOD plot.

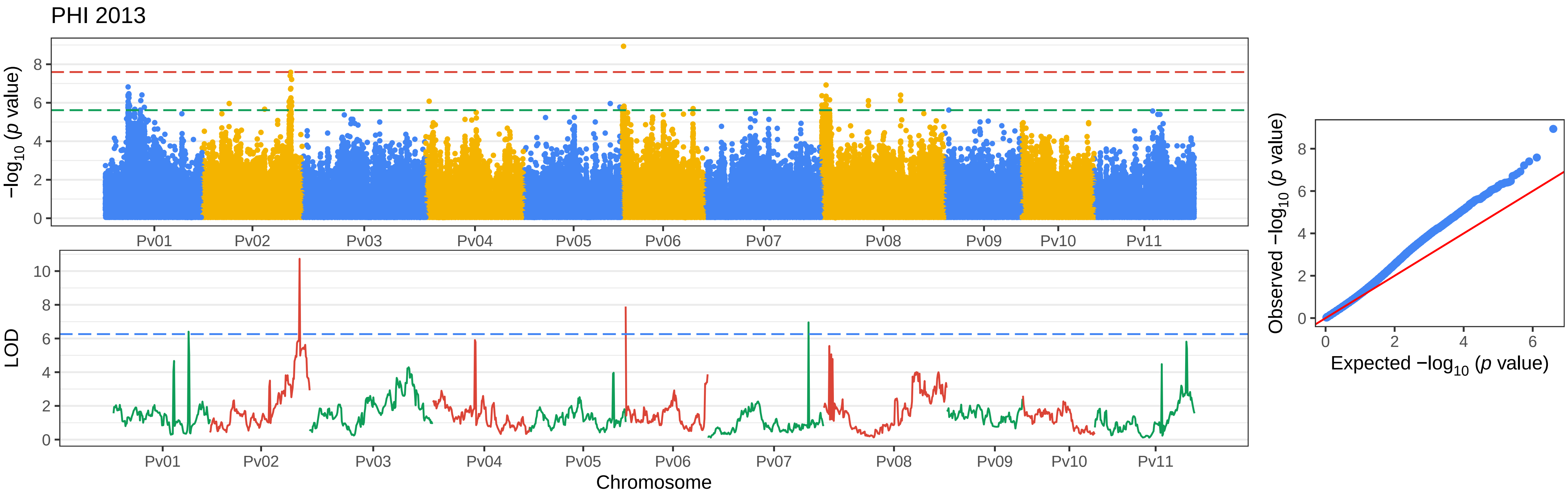


**Additional file 12.** Manhattan, quantile−quantile and LOD plots of the association and linkage mapping for each of the evaluated traits. The Bonferroni correction threshold ($p$ = 0.05) using the WGS (1,972,528) and the GBS (20,615) markers are depicted as red and green horizontal dashed lines, respectively, in the Manhattan plot. The significance threshold for the QTL mapping analysis is depicted as a blue dashed line in the LOD plot.
