# Supplementary material for: Genetic mapping for agronomic traits in a MAGIC population of common bean (Phaseolus vulgaris L.) under drought conditions
Source: BMC Genomics. 2020 Nov 16;21:799. doi: 10.1186/s12864-020-07213-6 (PMC7670608; doi:10.1186/s12864-020-07213-6)
Supplement: Supplementary file 14 — Additional file 14. Haplotype composition per chromosome of the top 10 RILs of the common bean MAGIC population. [file 12864_2020_7213_MOESM14_ESM.pdf]

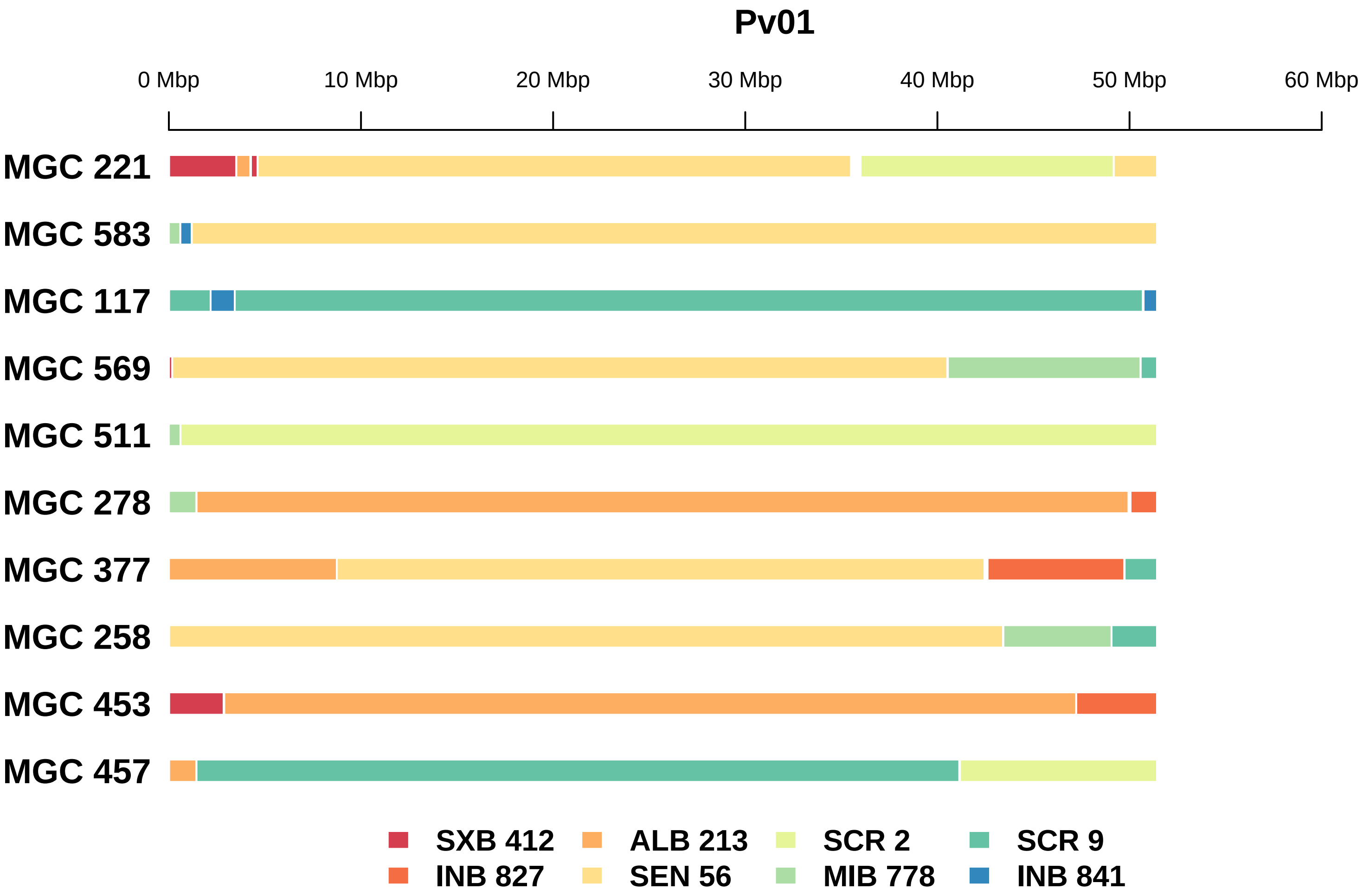

Additional file 14. Haplotype composition per chromosome for the top 10 RILs of the common bean MAGIC population.

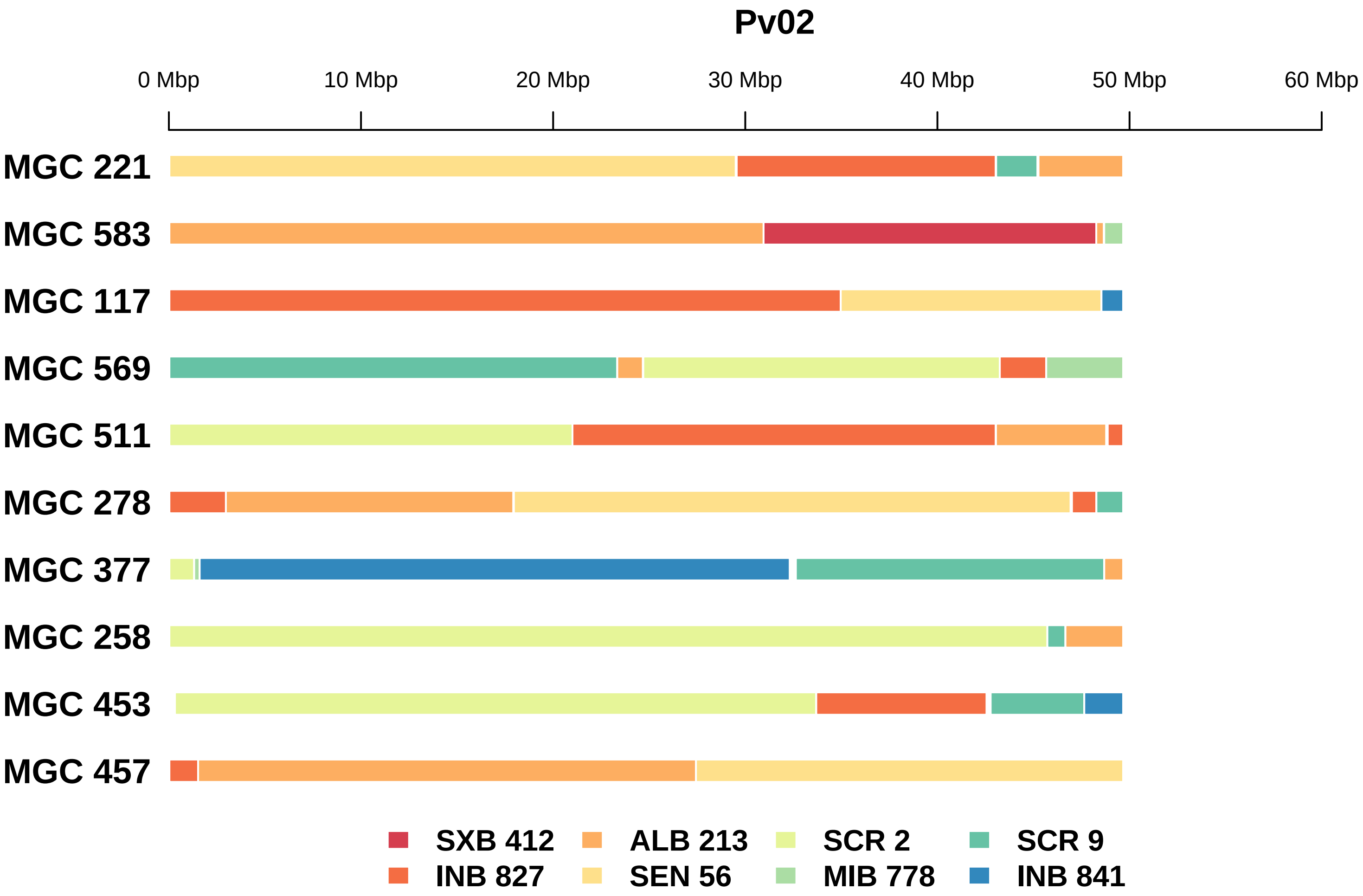

Additional file 14. Haplotype composition per chromosome for the top 10 RILs of the common bean MAGIC population.

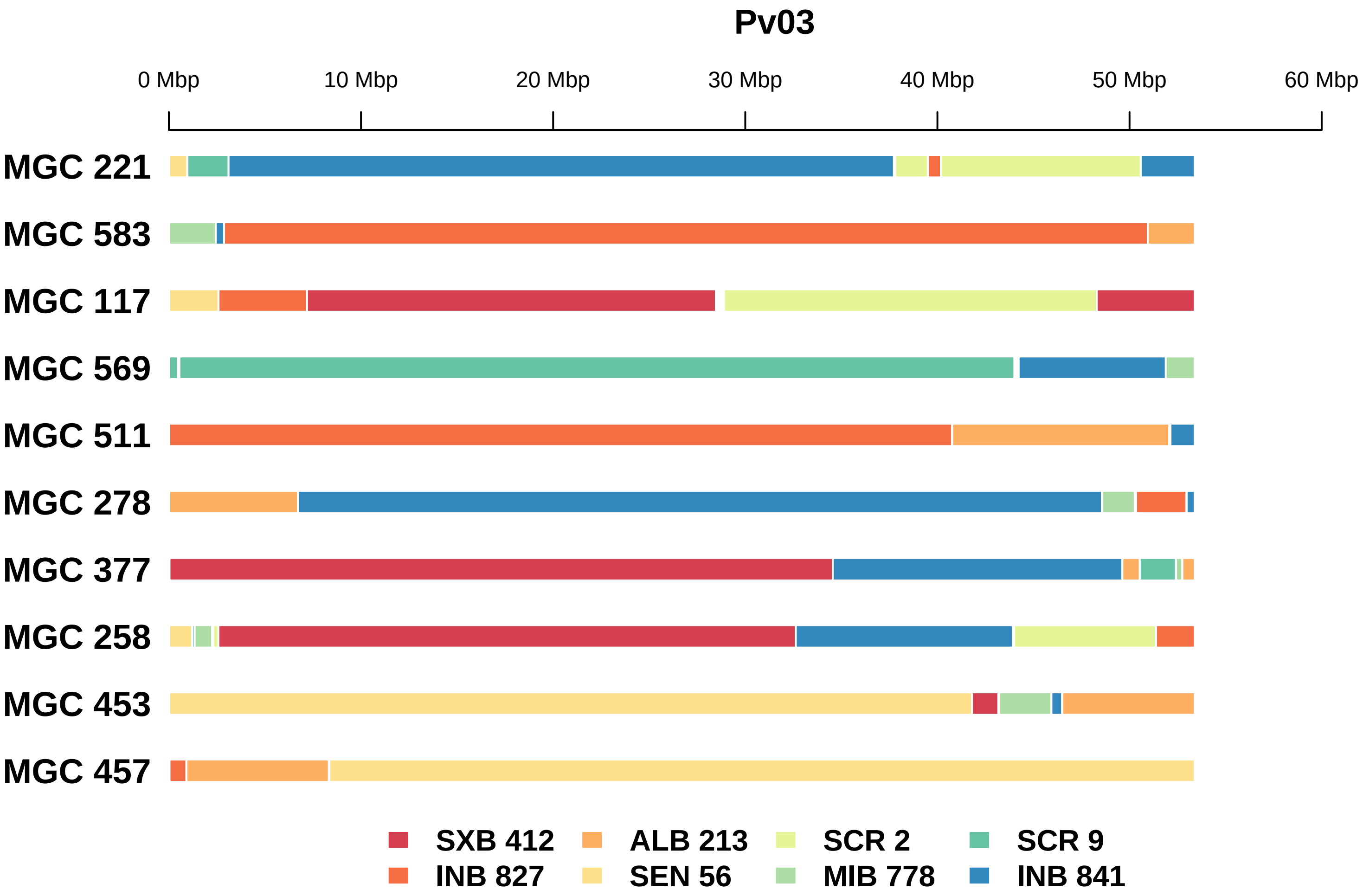

Additional file 14. Haplotype composition per chromosome for the top 10 RILs of the common bean MAGIC population.

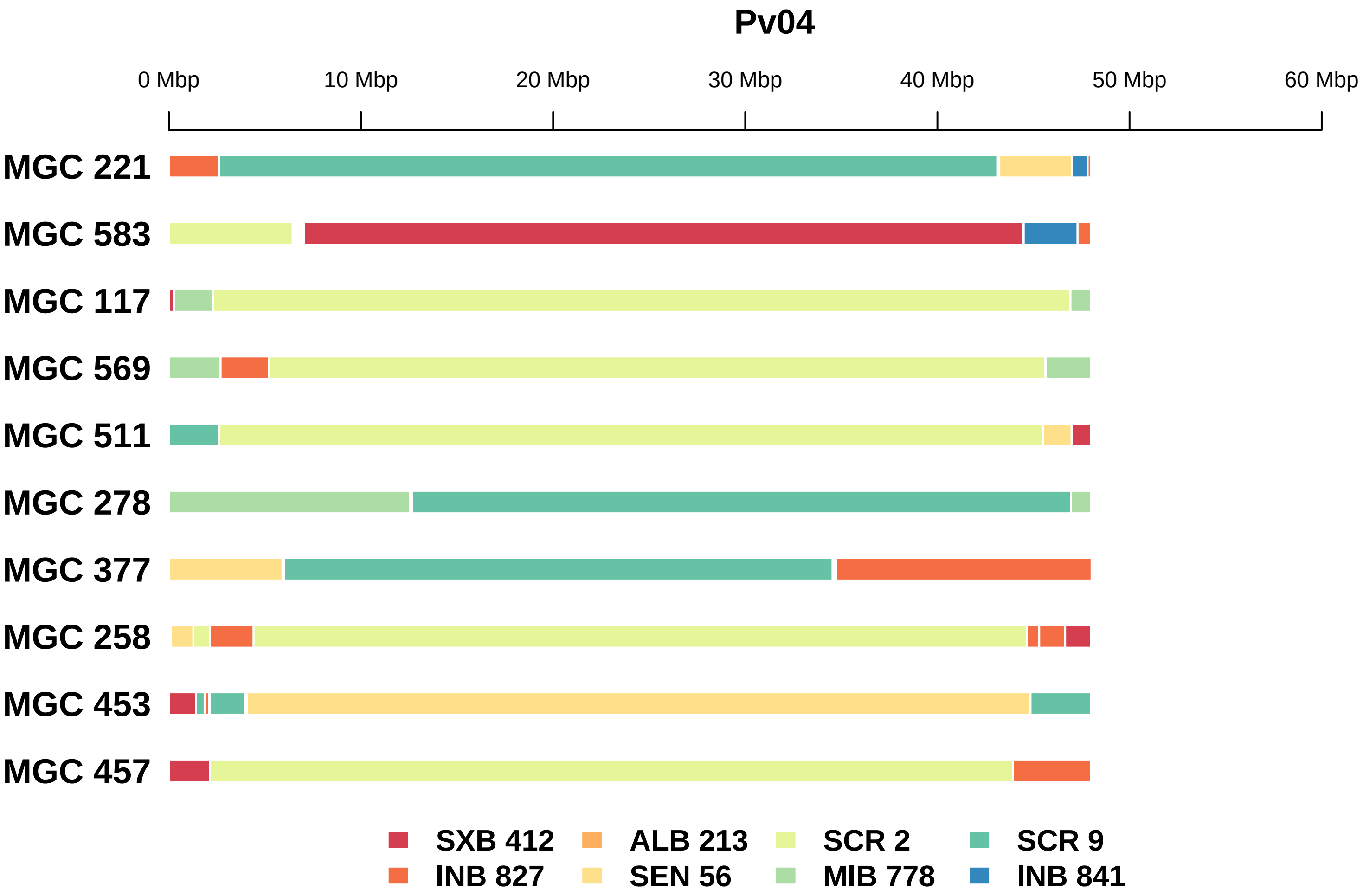

Additional file 14. Haplotype composition per chromosome for the top 10 RILs of the common bean MAGIC population.

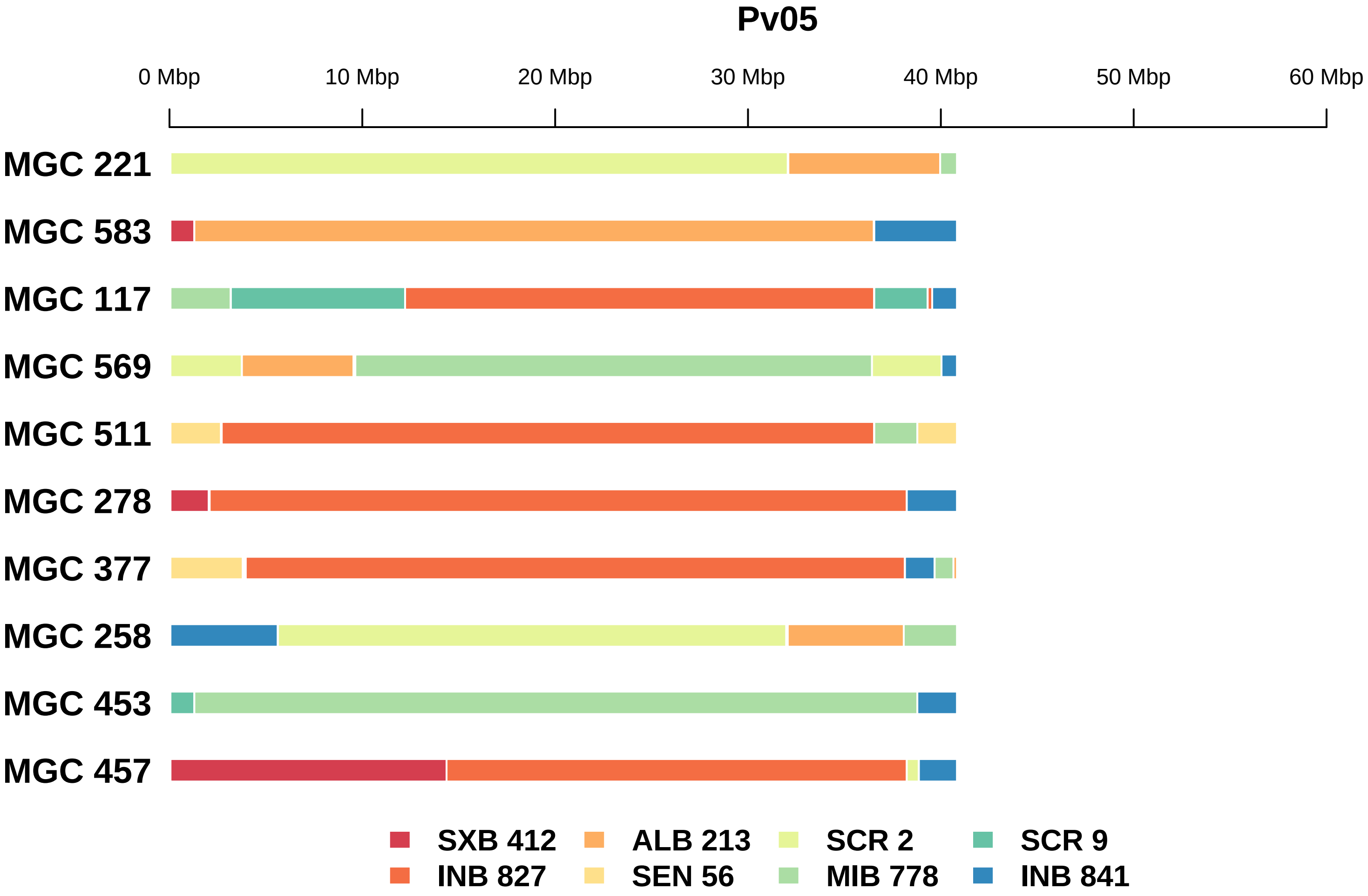

Additional file 14. Haplotype composition per chromosome for the top 10 RILs of the common bean MAGIC population.

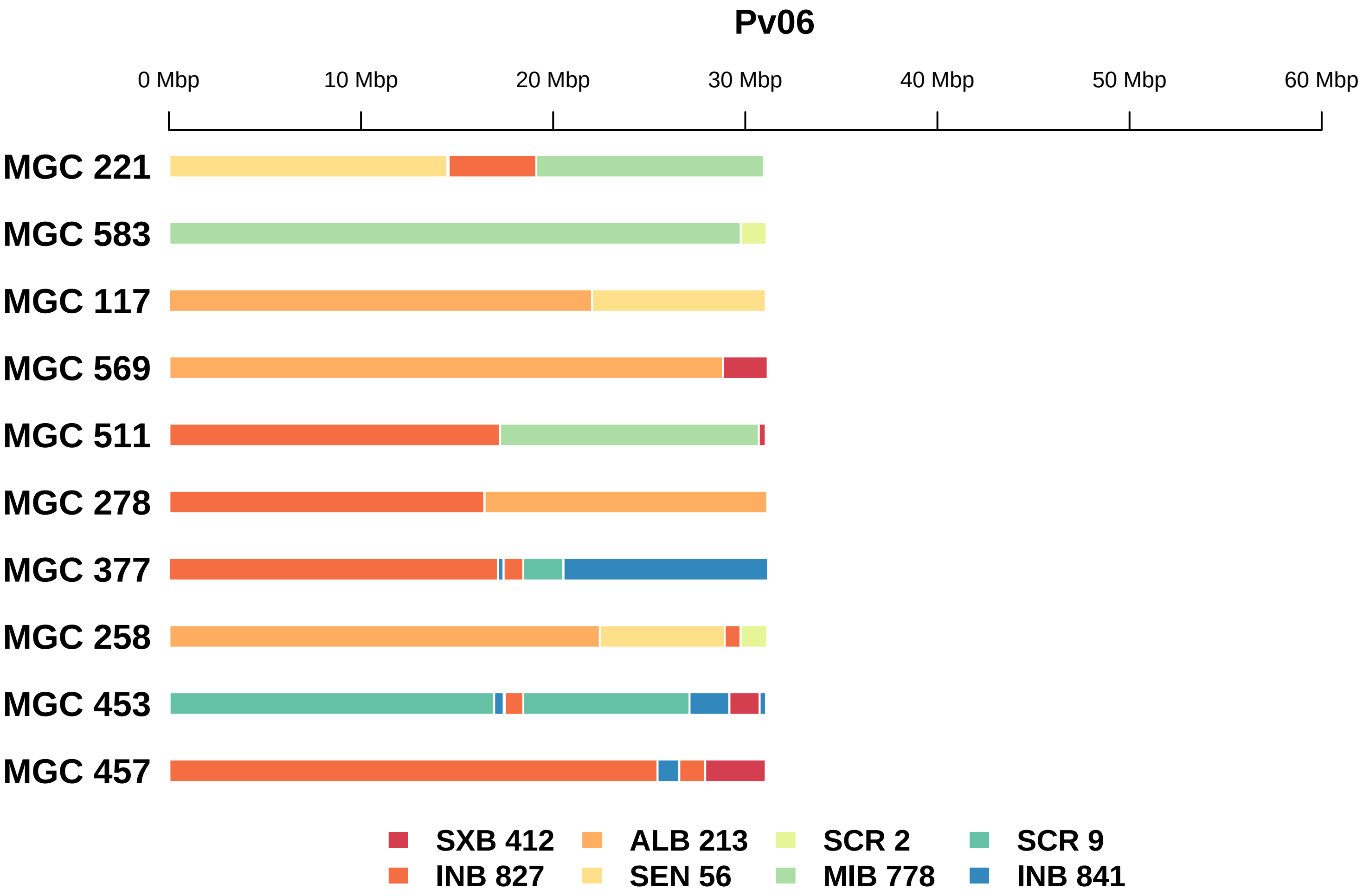

Additional file 14. Haplotype composition per chromosome for the top 10 RILs of the common bean MAGIC population.

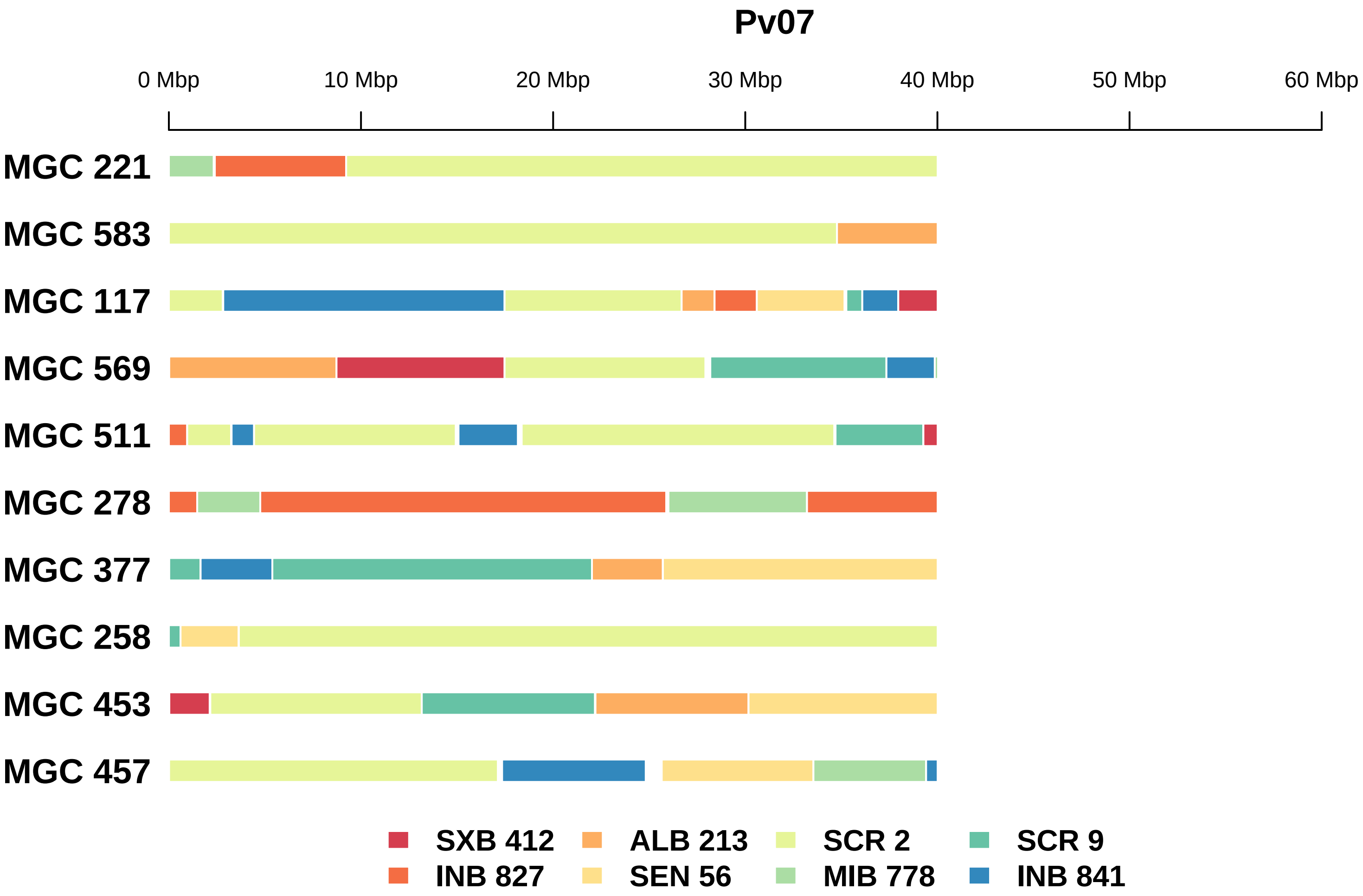

Additional file 14. Haplotype composition per chromosome for the top 10 RILs of the common bean MAGIC population.

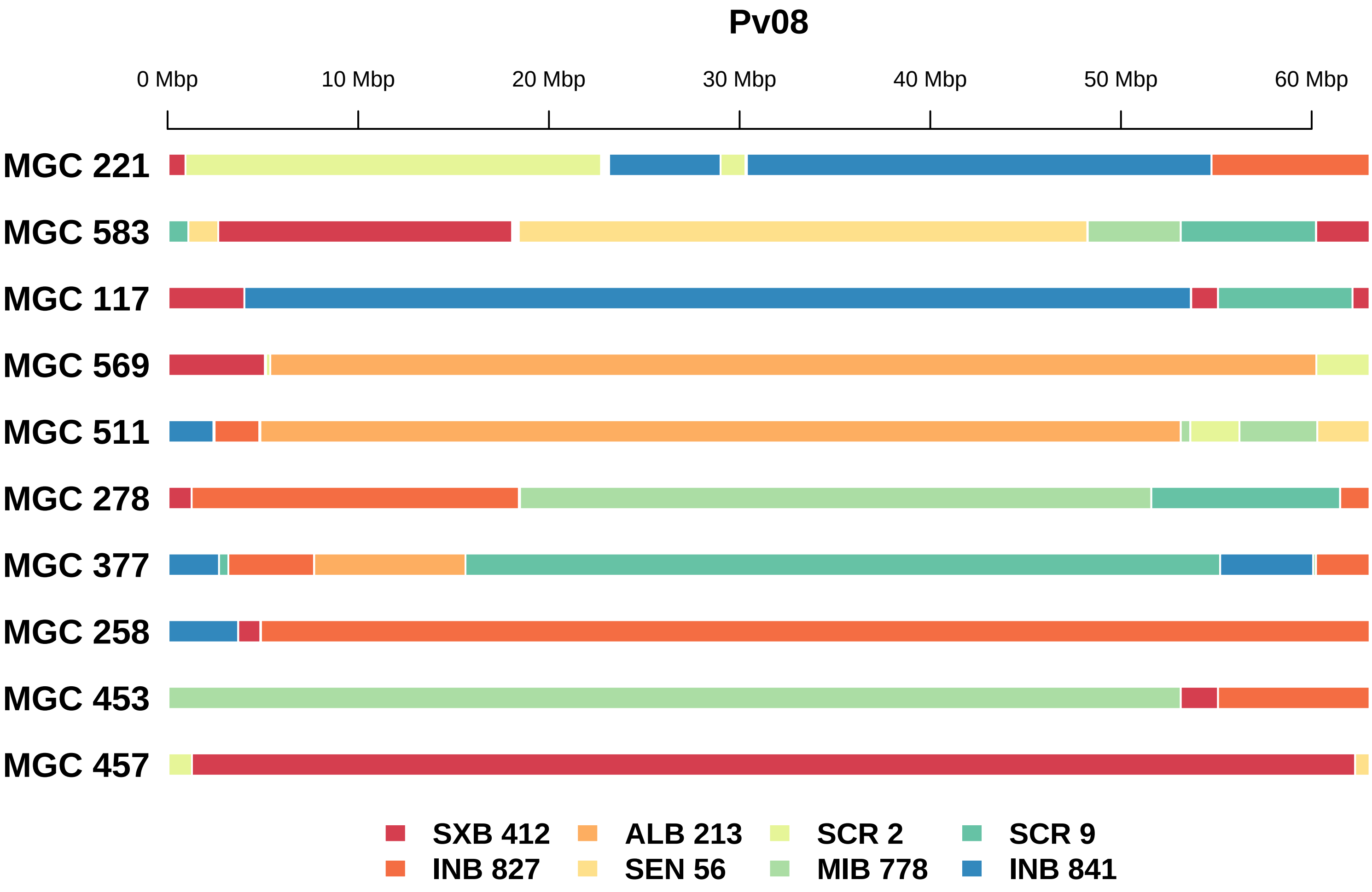

Additional file 14. Haplotype composition per chromosome for the top 10 RILs of the common bean MAGIC population.

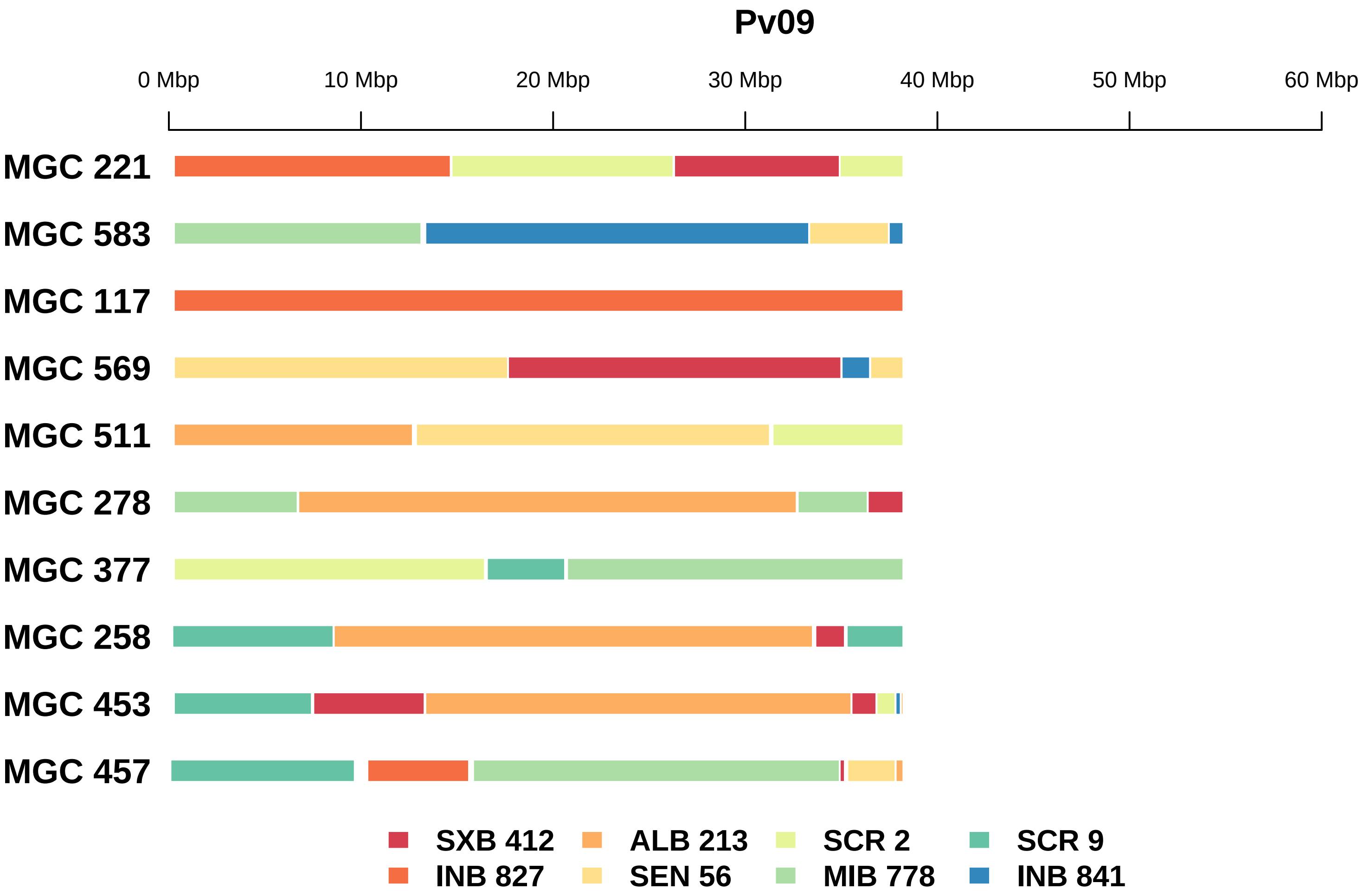

Additional file 14. Haplotype composition per chromosome for the top 10 RILs of the common bean MAGIC population.

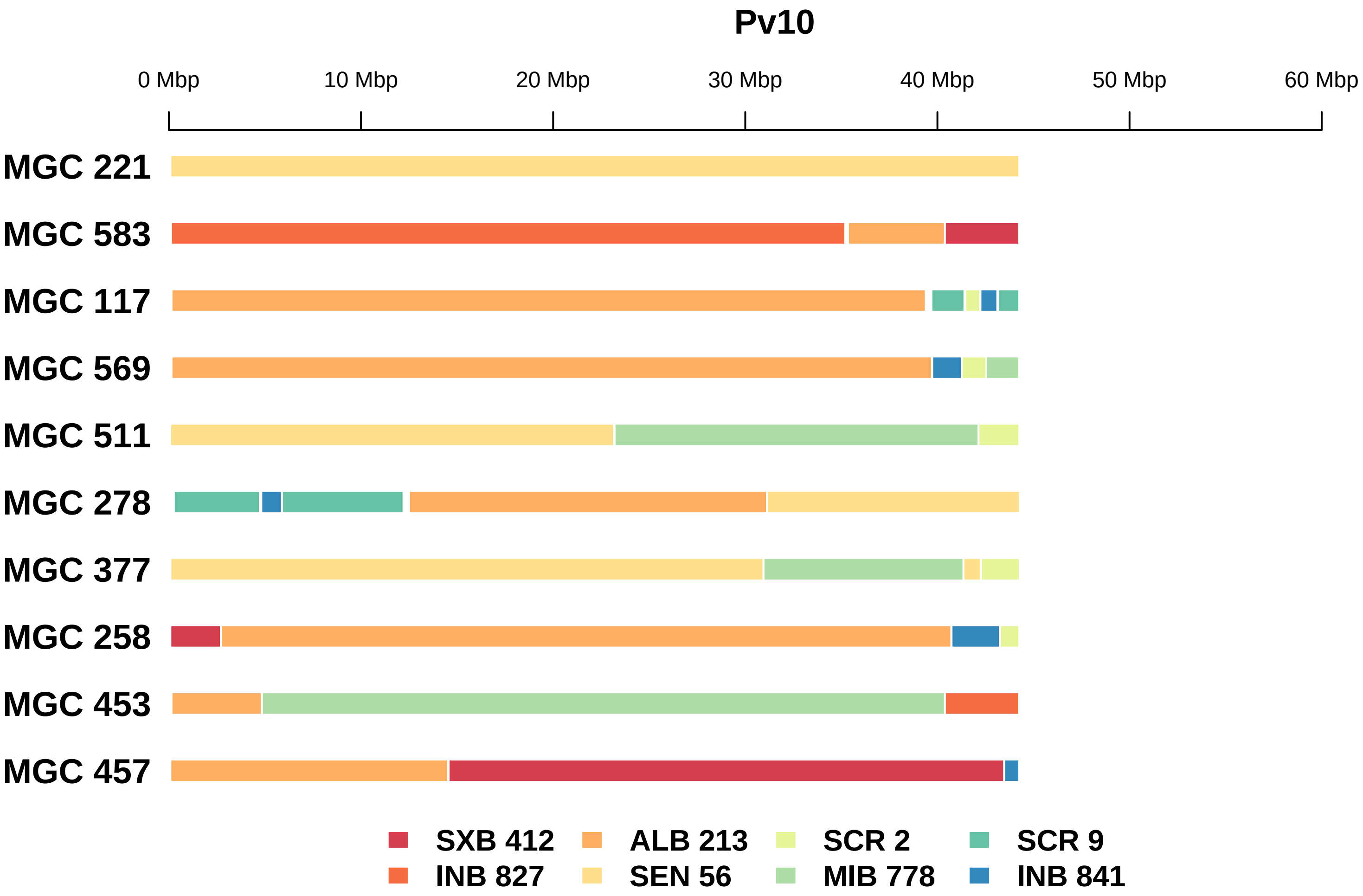

Additional file 14. Haplotype composition per chromosome for the top 10 RILs of the common bean MAGIC population.

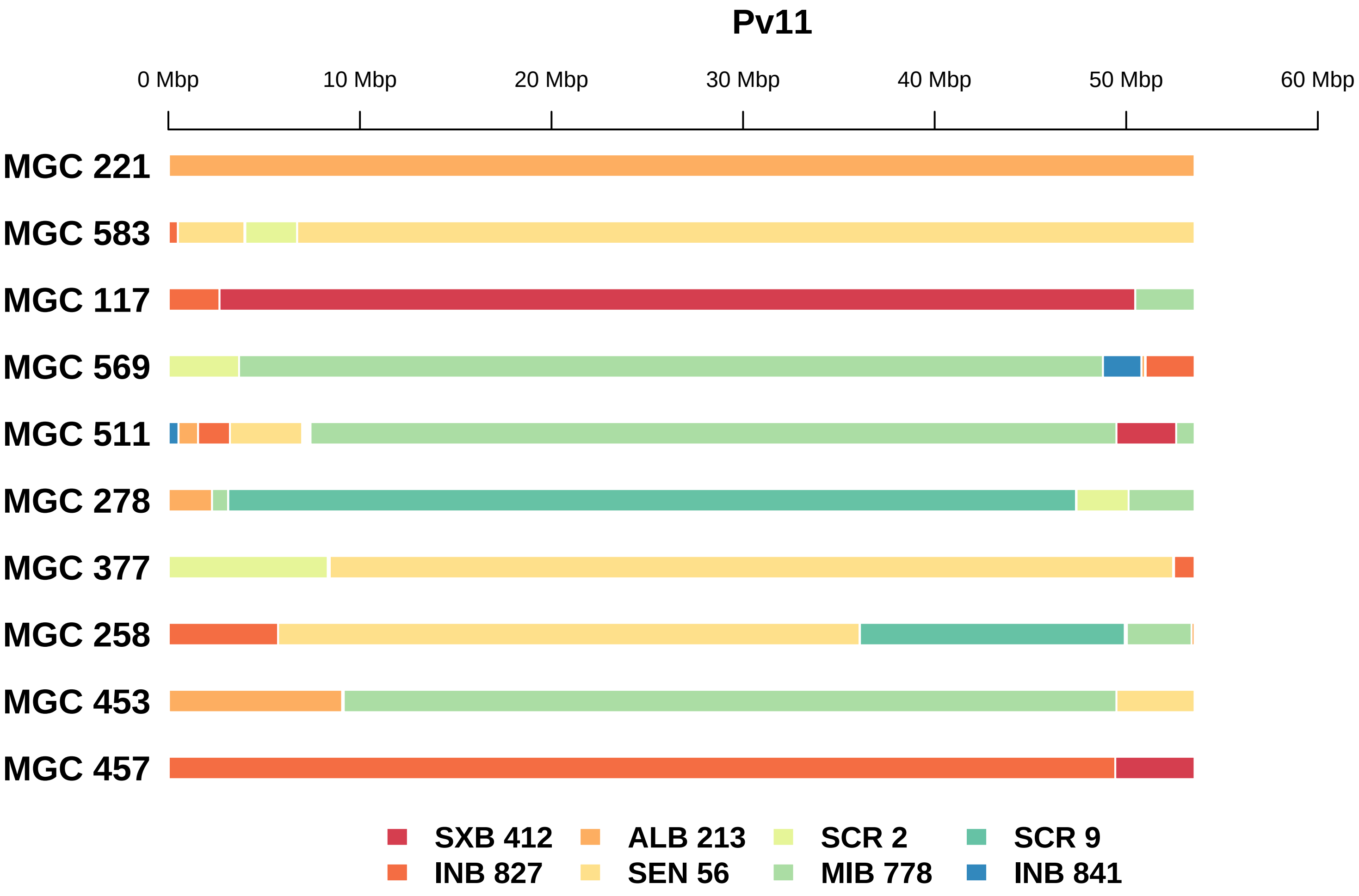

Additional file 14. Haplotype composition per chromosome for the top 10 RILs of the common bean MAGIC population.
